# Identification and Quantification of Multiphase U(VI) Speciation on Gibbsite with pH Using TRLFS and PARAFAC of Excitation Emission Matrices

**DOI:** 10.1021/acs.est.4c06133

**Published:** 2024-09-24

**Authors:** Laura Lopez-Odriozola, Samuel Shaw, Liam Abrahamsen-Mills, Charlotte Waters, Louise S. Natrajan

**Affiliations:** † Centre for Radiochemistry Research, Department of Chemistry, The University of Manchester, Manchester M13 9PL, U.K.; ‡ Research Centre for Radwaste Disposal and Williamson Research Centre for Molecular Environmental Science, Department of Earth and Environmental Sciences, The University of Manchester, Manchester M13 9PL, U.K.; § National Nuclear Laboratory, Warrington, WA3 6AE Cheshire, U.K.

**Keywords:** PARAFAC, TRLFS, uranium, speciation

## Abstract

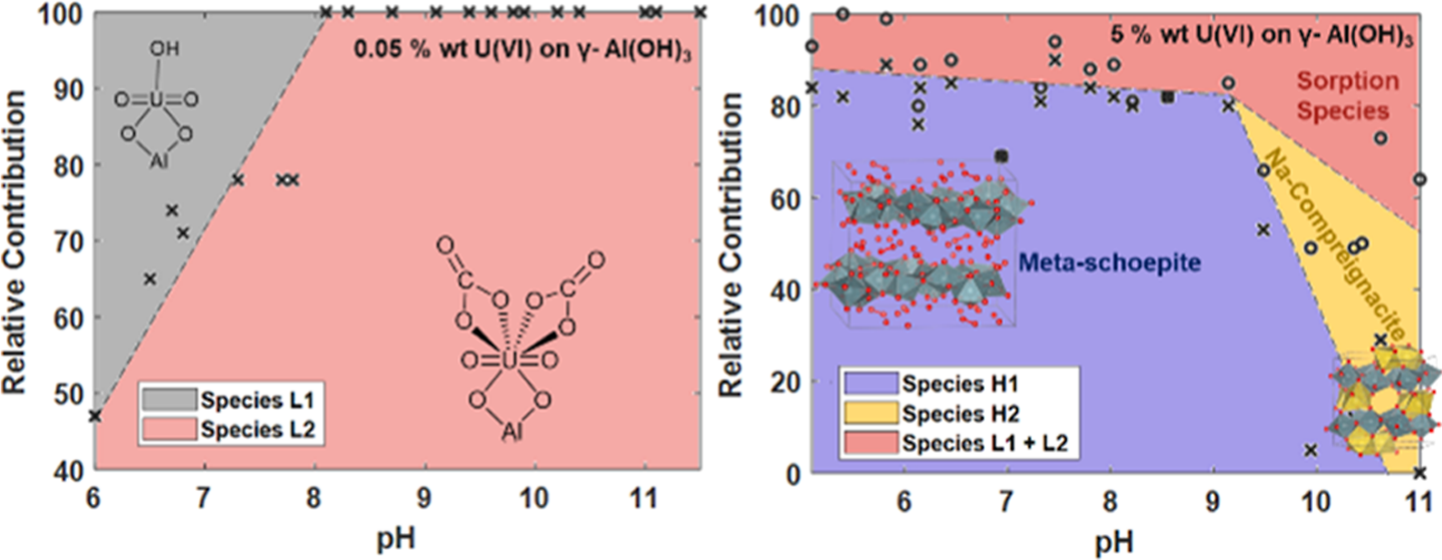

The significant abundance of uranium in radioactive waste inventories worldwide necessitates a thorough understanding of its behavior. In this work, the speciation of uranyl(VI), (UO_2_^2+^) in a gibbsite system under ambient conditions has been determined as a function of pH by deconvolution and analysis of luminescence spectroscopic data. Uniquely, a combined experimental and statistical approach utilizing time-resolved luminescence spectroscopy and parallel factor analysis (PARAFAC) of excitation emission matrices has been successfully utilized to identify four separate luminescent U(VI) species in the uranyl-gibbsite system for the first time. The speciation of all luminescent U(VI) species in an environmentally relevant system over a pH range of 6–11 is discerned through the analysis of emission fingerprints at low temperature (20 K). Comparison of the deconvoluted luminescence spectra with mineral standards and geochemical models of the system allows the assignment of the luminescent chemical species as metaschoepite, Na-compreignacite, surface adsorbed ≡AlO_2_–UO_2_(OH) and ≡AlO_2_–UO_2_(CO_3_)_2_^4–^ complexes, with assignments supported by fitting of extended X-ray absorption fine structure data. The combined spectroscopic techniques in this study show that assignment and quantification of uranyl(VI) species in a sorption system over a large pH range can be accurately achieved using PARAFAC to deconvolute a three way emission spectroscopic data set.

## Abstract 2

U(VI) phases precipitated and adsorbed on gibbsite are elucidated through mathematical deconvolution of luminescence emission data.

## Introduction

Uranium is the most abundant radionuclide by mass in radioactive waste inventories worldwide, for example it constitutes 95% of the UK’s nuclear materials by mass,^1^ and as such is an potential radio- and chemo-toxic environmental contaminant in a multitude of scenarios. These include radioactive contaminated land and surface to geological disposal. Understanding the speciation in such scenarios under relevant conditions is crucial for predicting the mobility and fate of uranium; and is therefore essential for making decisions regarding the disposal of radioactive wastes. Furthermore, uranium is found as the highly mobile uranyl(VI), UO_2_^2+^, ion in oxic aqueous conditions, which is known to complex with ions in solution (e.g., CO_3_^2–^), to adsorb to a number of materials (e.g iron oxides and zeolites) and minerals, as well as precipitate under certain conditions.^2−4^ Gibbsite, γ-Al(OH)_3_, is a mineral of key interest; as it is formed within some soil environments (e.g., tropical soils) and often used as a phase representative of the aluminum hydroxide layer in clay minerals, which are ubiquitous in near surface soils and sediments. Due to the proclivity of U(VI) to interact with available complexants and materials, uranium is often present as a number of chemical species (e.g., aqueous, adsorbed species and U-mineral phases) in one system (e.g., soil sample), and these need to be identified and quantified in order to develop a holistic understanding of uranyl mobility and fate in key environmental situations.^5^

Resolving U(VI) speciation is often a complex undertaking. Bulk spectroscopic techniques can struggle to separate signals from different species and quantifying contributions can be challenging, requiring multiple approaches and techniques. The characteristic, and highly informative, luminescence of the uranyl(VI) moiety has been utilized in a number of areas, such as chemistry, mineralogy and biology, and is a useful tool for determining the bonding environment of the ion and thus providing information on chemical speciation.^6−11^ Coupling of the photoexcited electronic state of uranyl to the total symmetric stretch (ν_1_) of the O=U=O bond results in a characteristic vibrational progression in the emission spectrum. This attribute has been used to identify molecular species in many systems ranging from biological, aqueous, and mineral to determine uranyl speciation.^7,9,12−17^ However, problems in analysis arise when systems contain multiple overlapping signals originating from different species, which is often due to the well documented complex and multiphase (e.g., mixtures of adsorbed species and solid phases) speciation of uranyl in natural and engineered environments. This is further complicated by the presence of additional environmental complexants, and spectral line broadening and quenching of the uranyl(VI) luminescence which occurs at room temperature.

Time resolved laser fluorescence spectroscopy (TRLFS) has been demonstrated to be a useful tool for the deconvolution of luminescent uranyl species in solution and/or sorbed to mineral phases that possess differing spectral and temporal profiles (luminescence lifetimes). However, it is often not effective in separating species in systems containing multiple components or chemically similar species with alike spectral profiles and luminescence lifetimes. Other deconvolution tools include principal component analysis and parallel factor analysis (PARAFAC), which can be effective alternative methods for the deconvolution of both time-resolved and steady state luminescence signals, in sample sets with differing lifetimes and excitation profiles, respectively. PARAFAC in particular has been demonstrated to be of value when applied to time-resolved emission spectra (TRES) or excitation emission maps [excitation emission matrice (EEM)]in a number of fluorophore containing systems and in aqueous uranyl systems.^18−22^ PARAFAC, however, has not yet been applied to systems containing solid uranyl phases. As well as preserving the speciation in the system during measurements, cryogenic temperatures have been shown to be efficient in enhancing luminescence signals by inhibiting the well-known vibrational quenching of uranyl luminescence that occurs at ambient temperatures. Furthermore, low temperatures reduce non-Boltzmann population of states, minimizing line broadening and yielding emission spectra with greater vibrational resolution.

A number of studies investigating uranyl sorption to gibbsite have been documented; X-ray absorption spectroscopy (XAS), TRLFS and computational methods such as density functional theory (DFT) have been successfully used individually and together to aid in elucidating uranyl speciation associated with gibbsite. From these, uranyl has been proposed to bind to the gibbsite surface via aluminol (≡AlOH) sites as a mononuclear inner-sphere surface species, ≡AlO(UO_2_)^+^, below pH 5.^23−25^ Above pH 6, this species is found alongside polymeric species, the assignment of which varies between existing studies; the formation of a carbonate complex, (UO_2_)_2_(CO_3_)(OH)_3_^–^, a solid precipitate and a polynuclear uranyl hydroxide have all been proposed.^23,26,27^ The carbonate-free system has been better characterized, with four luminescent species, assigned as, ≡AlO(UO_2_)^+^, ≡Al(UO_2_OH), electrostatically adsorbed UO_2_^2+^, and a solid metaschoepite ((UO_2_)_8_O_2_(OH)_12·_10(H_2_O)) precipitate, have been identified between pH 4 and 8 at ionic strengths of 0.0001, 0.1, and 0.4 M.^24^ Furthermore, while some of this binding is proposed to be monodentate, DFT calculations have indicated bidentate binding to the gibbsite surface is favorable.^25,28,29^ As well as the identity of adsorbed species, the pH and solution composition of a system will inevitably have consequences for the stability of any solid U(VI) phase which may form and as such it is important to consider the stability of relevant U(VI)-bearing solids, such as metaschoepite, compreignacite and uranate phases. Studies show metaschoepite and Na-compreignacite (Na_2_(UO_2_)_6_O_4_(OH)_6_·7H_2_O) are both least soluble between pH values of 6 and 8,^30,31^ and compreignacite and uranate phases have limited solubility at alkaline pHs.^32,33^ While in a number of existing studies, there is a distinct focus on low U(VI) loadings and CO_2_-free systems, the surface storage of U(VI) containing wastes and remediation of radioactive contaminated lands provide a rationale for further investigation of the system under oxygenated ambient conditions.

A thorough understanding of the interactions between U(VI) and gibbsite is crucial for evaluating U migratory and retention behavior with ubiquitous mineral phases, and therefore its fate in the environment. Here, TRES and EEMs recorded at cryogenic temperatures are utilized in tandem to identify uranyl speciation in a gibbsite containing system as a function of pH. Two loadings of uranyl on gibbsite are used in this study, a low (25 μM U(VI)) and high loading (HL) (2 mM U(VI)), in a pH range encompassing circumneutral to alkaline conditions (pH 6–11). For the first time, we demonstrate the utility of PARAFAC on the EEMs in a HL regime and show that when the spectra obtained are considered and validated alongside the species identified from lifetime analysis of TRES data acquired from a low loading (LL) regime, four individual luminescent U(VI) species with distinct emission spectra can be identified and quantified in a complex multiphase system containing both solid and adsorbed species.

## Methods

### Solid Phase Synthesis

Solid phases were synthesized as described in Section S1 of the Supporting Information.

### Sorption Experiments

In the HL samples, 10 mL of a 2.03 mM solution of UO_2_(NO_3_)_2_ in 0.1 M NaNO_3_ was agitated with 0.1 g of the synthesized gibbsite (S/L: 100 g/L) in a 15 mL centrifuge tube for each sample. The pH was adjusted to a set value between 5.1 and 11 twice a day for 3 days using 0.1 M NaOH and equilibration with air allowed. After this time the sample was left to equilibrate for 24 h, for a total contact time of 4 days. The final pH was recorded and the solid (as a paste) separated from the supernatant by centrifuging at 6500 rpm for 10 min. The quantity of uranyl in the solution before and after sorption was determined using colorimetric analysis with 2-(5-bromo-2-pyridylazo)-5-diethylaminophenol (Br-PADAP) with a limit of detection of 0.25 ppm U(VI) (1 μM).^34^ For LL samples, the procedure was repeated with a solution of 25 μM UO_2_(NO_3_)_2_ in 0.1 M NaNO_3_ in the pH range of 6 to 11 and the quantity of U(VI) in the solutions determined by ICP–MS due to its lower limit of detection.

### Luminescence Spectroscopy and Analysis

Samples were prepared for luminescence analysis by drop deposition of the gibbsite-uranyl paste on a fused silica slide. This was left to dry overnight before loading into the sample chamber. All luminescence spectra were recorded at 20 K using an Edinburgh Instruments FLS-1000 Photoluminescence Spectrometer with a cryostat attachment and a Hamamatsu red sensitive (PMT-900) detector. Steady state EEMs were measured using a 450 W Xe lamp with a 455 nm long-pass filter on the detection arm. The excitation range was set between 250 and 430 nm in 5 nm steps, emission spectra were recorded in triplicate between 450 and 700 nm in 1 nm steps with a 0.1 s dwell time. All spectra were corrected in the excitation and emission mode using the detector correction files (in built to the Edinburgh Instruments software) to account for variations in the excitation lamp profile and detector sensitivity across the visible spectrum. TRES were collected using a microsecond flash lamp (100 Hz, 0.1 ms delay) at an excitation wavelength of 285 nm. 8000 channels over 2 ms were used to record time-resolved emission intensity from 460 to 650 nm in 2 nm steps. A bandwidth of 1 nm was used in both the excitation and emission modes for time-resolved measurements, with a 455 nm long-pass filter in the detection arm to avoid any second order effects. MATLAB 2021a was used for analysis of all luminescence data.

The N-Way toolbox 3.30^35^ which contains all the necessary functions and scripts, was used to implement PARAFAC on the EEMs and carry out the deconvolution. Raw data were normalized to a maximum of one in the emission mode to prevent any single sample from contributing to the model in a disproportionate manner and non-negativity constraints were used in all modes. Note that for the LL data set, PARAFAC was attempted, but the model did not converge most likely due to the excitation spectra exhibiting maxima that are too similar in energy resulting in nonlinearity in the PARAFAC analysis. For this reason, time-resolved emission spectroscopy was employed to separate out the different species with a common excitation wavelength using lifetime differences. Gaussian deconvolution of emission spectra in a wavenumber scale was carried out by least-squares fitting of the data with initial guessing parameters set to the observable peak positions in the spectra. The symmetric vibrational stretches of the U=O bond, ν_1_, reported in this work have been calculated by averaging the spacing between the well resolved bands in the emission spectra.

### X-ray Absorption Spectroscopy

Samples from the two sorption sets were prepared for XAS analysis; three samples in the HL set at pH 5.5, 7, and 11 were mounted in cryogenic cells, frozen at −80 °C and analyzed at Diamond Light Source. A liquid nitrogen cryostat at beamline B18 (36-element Ge detector) was utilized to collect data at the U L_III_-edge in the transmission mode. The data were analyzed using the Demeter software packages Athena and Artemis, FEFF6, with the data typically fit to k and r space ranges shown in the Supporting Information.^36^

### Geochemical Modeling

PHREEQC^37^ geochemical software was used with the ThermoChimie (Debye–Huckle) v11^38^ database to predict the behavior of uranyl in the gibbsite system from existing thermodynamic data. Additional surface data from Karamalidis and Dzombak,^39^ which refer to a Diffuse Double Layer Model, was used to model the gibbsite surface, including binding constants of uranyl (≡AlO–UO_2_^+^) and uranyl hydroxides (≡AlO–UO_2_(OH)_3_^2–^, ≡AlO–(UO_2_)_3_(OH)_5_) and the carbonate (≡AlCO_3_^–^) binding constant estimated by Karamalidis and Dzombak from the limited available data. Binding constants for uranyl carbonate species to the gibbsite surface are not available in the literature.

PHREEQC was used to equilibrate the 0.1 M NaNO_3_ solution with the gibbsite surface at varying U(VI) concentrations in a pH range of 3 to 13, the solution pH was adjusted with HNO_3_ or NaOH and system equilibrated with atmospheric CO_2_. A number of U(VI) solid phases were identified as having a positive saturation index and allowed to equilibrate within the model if their formation under the experimental conditions was feasible (i.e., formation could reasonably occur at room temperature in the time scale of a week). These phases included a number of schoepite-like phases (metaschoepite, UO_3_·0.9H_2_O and β-UO_2_(OH)_2_), sodium-compreignacite, sodium uranate and the carbonate phases UO_2_(CO_3_)_3_Na_4_ and rutherfordine (UO_2_CO_3_).

## Results and Discussion

### Sorption and Geochemical Modeling

**Figure 1:**
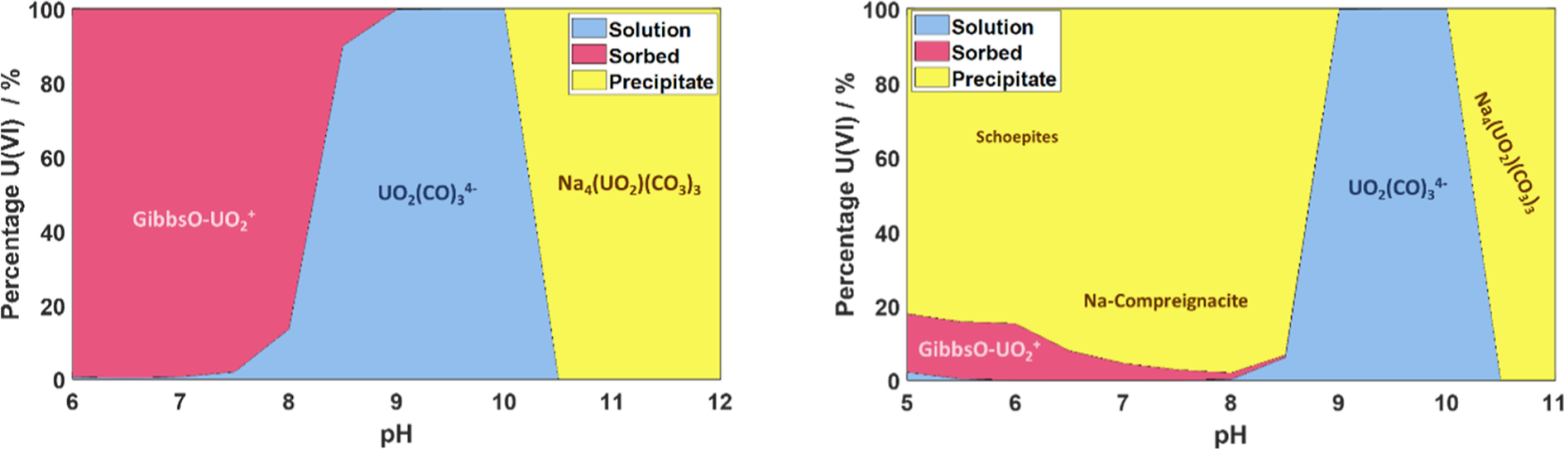
Area graphs showing most favorable form of U(VI) in LL (left) and HL (right) regime across the pH range from PHREEQC calculations.

Full or near full (>98%) removal of U(VI) from solution is observed above pH 6 at both concentrations of U(VI) studied (Figure S2). Geochemical modeling using available thermodynamic data at the two experimental U(VI) concentrations of 25 μM and 2 mM was carried out and the predicted speciation in the pH range is shown in Figure 1.

Below pH 9, sodium compreignacite and metaschoepite phases are predicted to be the major phases present in the system at higher U(VI) concentrations (2 mM). The sorbed species AlO–UO_2_^+^ is predicted to form below pH 9 and is the only significant surface complex present in the LL regime, up to 20% is predicted to form in the HL samples below pH 8. Despite the experimentally observed full removal of U(VI) from solution, the modeling results predict the aqueous uranyl(VI) tris-carbonate, UO_2_(CO_3_)^4–^, to be the dominant species in the system between pH 9 and 10 and thus it is evident the thermodynamic data does not accurately represent all processes in the system. However, this is likely due to the lack of available data for the adsorption of U-carbonate complexes to the gibbsite surface.

The inclusion of the gibbsite binding constants^39^ for the carbonate anion (CO_3_^2–^) predicts the surface to consist mostly of a combination of acidic hydroxide sites (Al–OH_2_^+^) and carbonate sites (Al–CO_3_^–^) in approximately equal proportions from pH 5 to 8.0. Above this pH, carbonate sites become preferable and by pH 10.0 virtually all surface sites are expected to be complexed by carbonate (Figure S3). As the complexation constants for the carbonate anion on the gibbsite surface are estimates based on linear free energy relationships rather than experimental results, calculations were also carried out without the inclusion of these, and no impact on the predicted uranium speciation or behavior was observed. The surface sites in this instance are predicted to be dominated by the neutral (Al–OH) site throughout the experimental range, with a third of the sites being acidic (Al–OH_2_^+^) at pH 5 and only a fifth of sites deprotonated (Al–O^–^) at pH 11.

### Luminescence Spectroscopic Analysis

**Figure 2:**
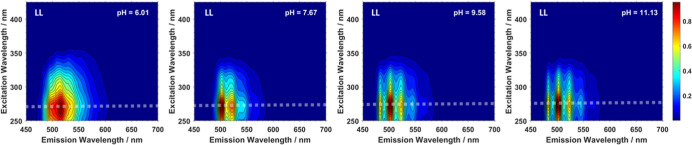
Normalized excitation emission maps of selected LL samples at pH (L-R) 6.0, 7.7, 9.6, and 11.1 recorded at 20 K, showing the constant excitation profile throughout the experimental range.

**Figure 3:**
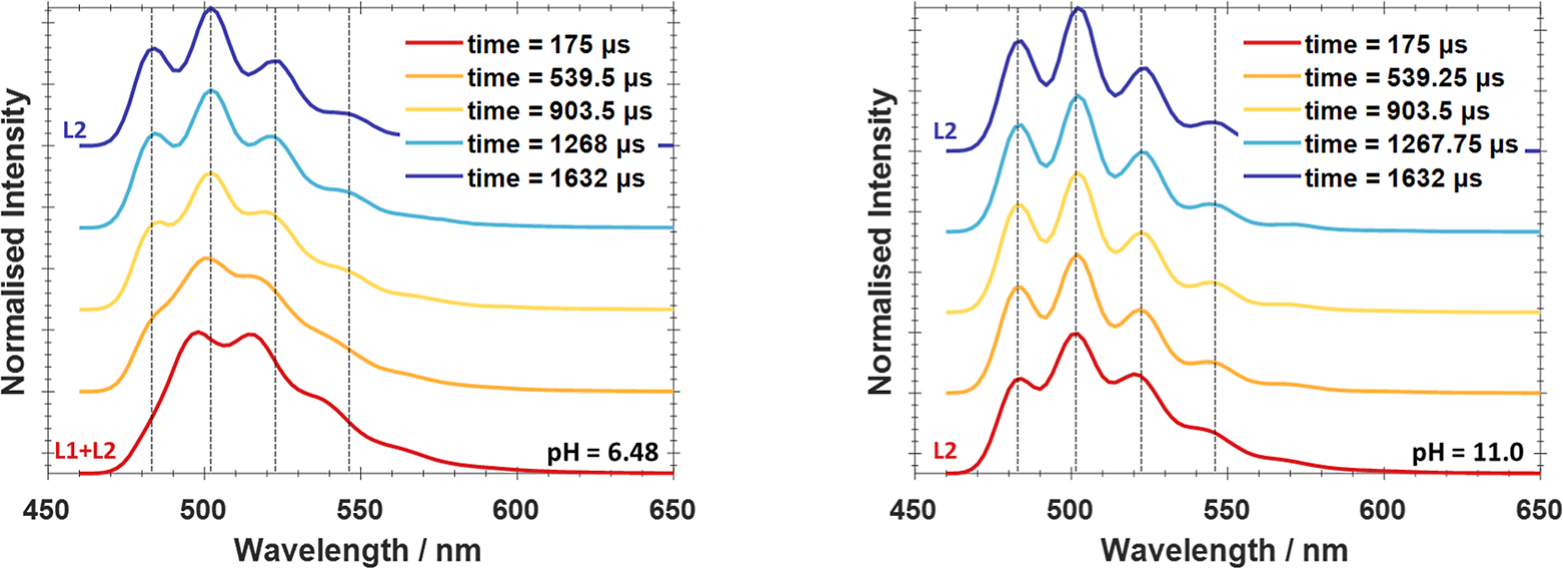
Normalized and stacked smoothed emission spectra of pH 6.48 (left) and pH 11.0 (right) samples, extracted from the TRES, recorded at 20 K using 285 nm excitation.

**Figure 4:**
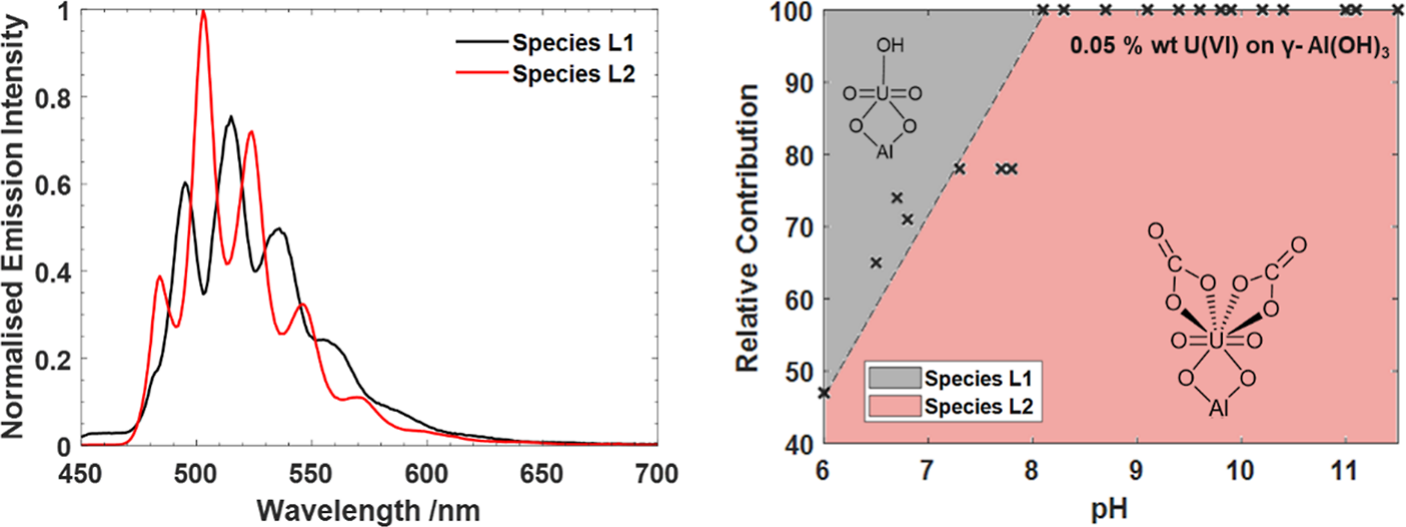
Emission spectra of the short and long-lived species after 285 nm excitation (left) and the contribution to the total luminescence of the samples across the pH range, with a straight dotted line indicating the approximate phase boundary (right). Sketches representing the assignments of the two species, discussed below have also been included.

The recorded EEMs of the LL sample set generally exhibit a constant excitation maximum throughout the pH range (Figures 2 and S4), at 275 nm, allowing deconvolution of species to be carried out by analysis of time-resolved spectra recorded at this wavelength. In the TRES of the LL system samples, shown in Figure S5a marked change in the emission profile over time can be observed in the first seven samples (pH 6–7.8), the emission profile becomes more resolved and a blue shift for the maximum intensity is observed. This suggest that multiple U(VI) species are present under these conditions. In the higher pH samples (pH 8–11), there are no changes in emission profile with respect to time, suggesting only one luminescent U(VI) species is present under these conditions, an example of the emission spectra extracted from TRES for two of the samples (pH 6.48 and 11.0) is shown in Figure 3. Lifetime fittings of the data indicated two major species and required the inclusion of a third shorter, minor component (accounting for <10% of the intensity); biexponential decay kinetics in lifetime fittings of single U(VI) species have been observed previously in the literature.^9,17,40,41^ The origin of this behavior has been postulated to be due to a redistribution of energy and small changes in local uranyl coordination environments. However, the precise origin of these observations remains unclear. The two species were ascertained to possess luminescence lifetimes of 230 ± 30 μs and 750 ± 50 μs. The emission spectra of the two moieties and their calculated contributions to the luminescence intensity through the experimental pH range of the LL system are shown in Figure 4. Species L1 the short-lived species, was found to have emission peaks at 495, 515, 535, 561, 584, and 612 nm and only contributed to the total luminescence intensity below pH 8. Species L2 is longer lived and found throughout the pH range, with emission maxima at 485, 503, 523, 546, 570, and 599 nm.

**Figure 5:**
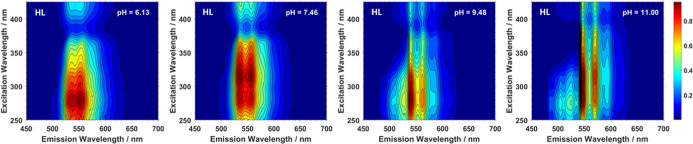
Normalized excitation emission maps of selected HL samples at pH (L-R) 6.1, 7.5, 9.5, and 11.0 recorded at 20 K, showing the lack of consistency in the excitation profile throughout the experimental range.

**Figure 6:**
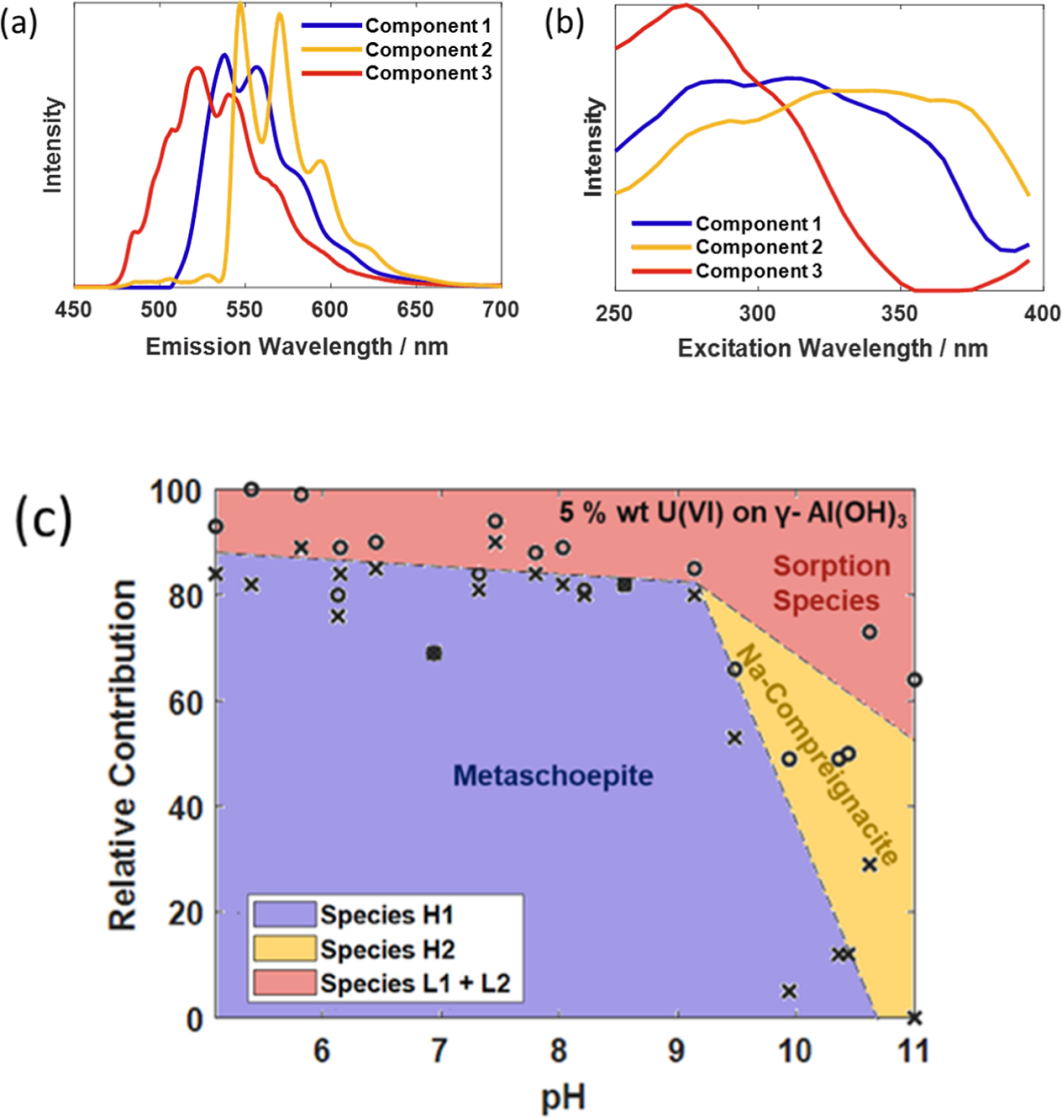
Results from PARAFAC of the HL set showing the deconvoluted (a) emission spectra (b), excitation spectra and (c) relative loadings for the three components. The boundaries between components determined experimentally are marked with black crosses (components 1/2) and open circles (components 2/3). Dashed lines have been added to indicate approximate phase boundaries and the assignments of the two species (discussed below) are included.

In the HL system, the emission spectra are more complex. Spectra are shown in Figure S6 and four examples in Figure 5. Both the steady state emission and excitation profiles vary considerably in both shape and band position across the pH range. The emission profile is more vibrationally resolved at alkaline pH values, where a greater intensity at higher energy is also generally observed. The excitation profile is broader than that observed previously at low UO_2_^2+^ loadings, and multiple maxima are observed in a number of the EEMs. The EEMs were normalized in the emission mode and processed using PARAFAC. A PARAFAC model (Figure 6) comprised of three components was identified as most suitable and was validated by split half analysis (Figure S12.).^42^ The emission spectra derived from the model are in accordance with emission spectra expected from uranyl(VI) species, which typically exhibit a defined vibronic progression.^2^

Components **1** and **2**, exhibit a clear vibrational progression in their emission profiles, as shown in Figure 6, suggesting each component corresponds to a single uranyl species, termed species **H1** and species **H2** respectively from here on. The peak positions of the emission bands were determined to be 537, 559, 581, 609, and 640 nm and 548, 570, 594, 617, and 652 nm for species **H1** and species **H2** respectively. The symmetric vibrational stretch of the U=O bond, ν_1_, was calculated to be 747 cm^–1^ for species **H1** and 702 cm^–1^ for species **H2**. Due to the lack of marked vibronic progression in component 3, peak positions and a vibrational bond force constant values could not be determined at this stage, it is, however, likely to be representative of one or more U(VI) gibbsite surface complexes, which will undoubtedly form in this system.

### Analysis and Assignments of the Emissive Species

Comparison of the distribution of the two species in the LL sample set to the predicted speciation from geochemical modeling and existing studies enables assignment of species **L1** to a uranyl center sorbed to an aluminol site on the gibbsite.^23−25^ This assignment is supported by comparison of the emission spectrum of species **L1** (peaks: 495, 515, 535, 561, 584, 612 nm; ν_1_:760 cm^–1^) to the peak positions (496, 516, 538, 562 nm) and vibrational stretch (760 cm^–1^) for the sorption species ≡AlO–UO_2_(OH) found above pH 6 by Chang et al.^24^ (Table 1). Comparison of the symmetric vibrational stretch, ν_1_, of species **L1** to aqueous UO_2_^2+^ (861 cm^–1^) at cryogenic temperatures shows a significantly smaller value for species **L1**, indicative of the weaker O=U=O bond in the sorption species, due to the interactions between the aluminol site and the uranyl moiety.

**Table 1:** Peak Positions, Luminescence Lifetimes (τ) and Symmetric Stretching Frequencies (ν_1_) Calculated From the Luminescence Data for Each Deconvoluted U(VI) Species, Synthesised Mineral Standards, and a Selection of Relevant Literature Species^a^

U(VI) species	peak positions/nm						ν_1_/cm^–1^	τ/μs	*T*/K	ref
species **L1**	495	515	535	561	584	612	760 (11)	230 (30)	20	this work
species **L2**	485	503	523	546	570	599	770 (14)	750 (50)	20	this work
species **H1**	537	559	581	609	640		750 (50)		20	this work
species **H2**	548	570	594	617	652		702 (2)		20	this work
Na-compreignacite	548	570	593	616	655		690 (6)		20	this work
metaschoepite	530	553	578	601	636		780 (3)		20	this work
K_3_Na(UO_2_)(CO_3_)_3_·H_2_O	474	492	512	534	562		779 (4)		20	this work
UO_2_^2+^ (aq)	491.7	513.9	538.4	563.5	591.9		861	270	6	(2)
UO_2_(CO_3_)_4_^3–^ (aq)	479.6	499.2	519.9	542.4	565.6		792	883,62	6	(2)
≡AlO–UO_2_(OH)	496	516	538	562			760 (21)		5	(24)
≡AlO–UO_2_^+^	494	514	537	562			820(22)		5	(24)

a The highest intensity peak for each spectrum is marked in bold.

The species **L2**, which dominates the **LL** system has a higher energy of emission and longer luminescence lifetime than species **L1**. This is consistent with it being a uranyl carbonate, as these have been demonstrated to emit at higher energies and possess longer lived photoexcited states at cryogenic temperatures than the uranyl aqua in solution. In particular, the emission energies for **L2** are broadly consistent with those of UO_2_(CO_3_)_4_^3–^_(aq)_, with a shift in peaks due to the change in coordination environment on adsoprtion (Table 1).^9,10,17^ Despite the proposed formation of polynuclear uranyl species on the gibbiste surface at pH 6–9 in previous studies,^25−27^ the proximity of additional uranyl moieties has been demonstrated to significantly broaden emission spectra.^8,43^ The distinct vibrational resolution observed here provides strong evidence for mononuclear uranyl sorption to the gibbsite surface. While we cannot conclude the exact nature of the binding, existing studies have demonstrated the bidentate binding of uranyl to the gibbsite surface is preferable,^27,28^ consequently species **L1** and **L2** are proposed to be two single uranyl centers bound in a bidentate manner; a uranyl hydroxide ≡AlO_2_–UO_2_(OH), and a uranyl carbonate, ≡AlO_2_–UO_2_(CO_3_)_2_^4–^.

**Figure 7:**
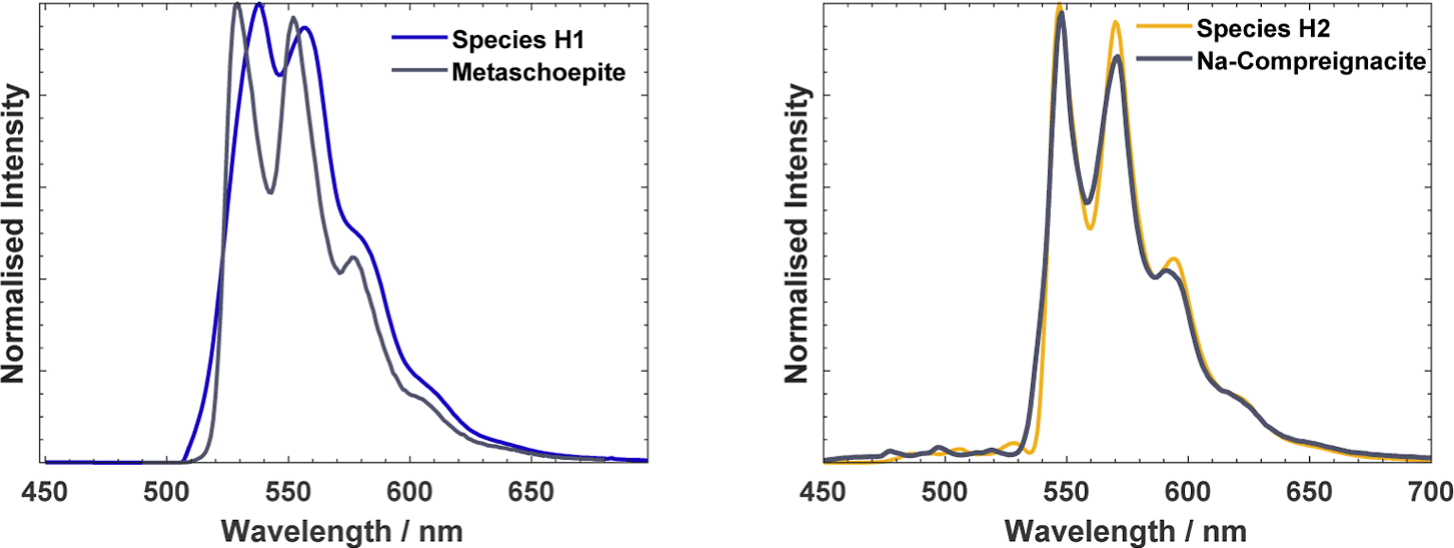
Emission spectra of the two mineral standards, metaschoepite (left) and Na-compreignacite (right), recorded at 20 K with an excitation source of 285 nm, with the corresponding species overlaid.

Three mineral standards, metaschoepite, sodium compreignacite and K_3_Na(UO_2_)(CO_3_)_3_·H_2_O were synthesized to aid in the assignment of species. The emission spectra of the solids were analyzed to determine the peak positions and the ν_1_ values calculated (Table 1). While we are limited by the minerals synthesized, comparison of ν_1_ and peak positions with the standards have enabled us to assign species **H1** and **H2**, obtained from components 1 and 2 in the PARAFAC deconvolution, as the two minerals, metaschoepite and sodium compreignacite (Figure 7). The metaschoepite standard has narrower peaks than species **H1** and is shifted to a slightly higher energy (smaller wavelength). This can be reasonably explained by the higher crystallinity expected from the mineral standard, as it was formed over a longer period of time. The lower crystallinity of metaschoepite formed in the gibbsite system will result in a greater distribution of energy states and consequently in a broadening of the emission signal. The ν_1_ values of **H1** is within that of the standard, supporting the assignment of species **H1** as metaschoepite.

Despite the predicted formation of Na_4_(UO_2_)(CO_3_)_3_ between pH 10 and 11 from geochemical modeling, comparison of **H2** with the emission spectra of a structurally analogous mineral (K_3_Na(UO_2_)(CO_3_)_3_·H_2_O, Figure S14) shows the lack of agreement between spectra. Comparison with the emission of a Na-compreignacite standard (Figure 7), however, shows negligible differences between these, allowing confident assignment of the species discerned by PARAFAC as the sodium analogue of the uranium mineral compreignacite. The formation of compreignacite relative to metaschoepite above pH 9 is consistent with studies of their relative solubility as a function of pH, and has indeed been observed previously on similar studies involving U(VI) interactions with hydrotalcite (Mg_6_Al_2_CO_3_(OH)_16_·4H_2_O), which has a similar surface structure to gibbsite.^30,33^

Despite the lack of distinct vibrational resolution of component 3, comparison to the emissive species **L1** and **L2** identified in the LL series suggests these two species can reasonably comprise component 3 (Figure S22). Furthermore, as evidenced by the EEMs recorded for the LL series the excitation spectrum for the two species overlap to form a broad spectrum centered around 275 nm. This is in accordance with the excitation spectrum discerned for component 3 from PARAFAC. The lack of uniqueness in the excitation mode explains why the two species could not be separated into two individual components using PARAFAC. The blue shift of component 3 relative to species **H1** and **H2** in both emission and excitation illustrates the larger HOMO–LUMO gap in the uranyl in these species. The higher energy indicates the equatorial ligands surrounding the uranyl moiety are weaker sigma donors than those of the solid phases,^44^ further supporting their assignment as sorption species.

Linear combination fits (LCF) of the L_3_-edge XANES were performed for the HL pH 5.5, 7, and 11.0 samples as shown in Figures S23 and S24. The data were fitted using the synthetic metaschoepite and Na-compreignacite standards. Standards for adsorbed species were not considered as the U(VI) local environment and hence the XANES spectra between U(VI)-uranyl adsorbed to a mineral surface is amost identical to that of U(VI) in schoepite. The LCF indicated the pH 5.5 and 7 samples are dominated by metaschoepite/adsorbed species. In contrast, Na-compreignacite dominates the pH 11 sample. This is broadly in agreement with the PARAFAC deconvolution results and the consequent species assignments, which show the contributions to the emission for the HL pH 5.5 and 7.0 samples to be approximately 80–90% metaschoepite/adsorbed species, with the remainder as sorption species. In the case of the pH 11 sample, 55% of the emission intensity is attributed to the presence of Na-compreignacite.

The assignments from luminescence data and LCF are further supported by the fitting result from the U L_3_-edge extended X-ray absorption fine structure (EXAFS) for the HL samples at the three pH values (5.5, 7.0, 11.0). The best fit parameters are shown in Table S4. The fits to the data from the HL samples at pH 5.5 and 7 indicate U in uranyl coordination, with a U–O_ax_ distance of 1.80 Å and 2 shells of U–O_eq_ at 2.26 (±0.01) and 2.41 (±0.01) Å. In addition, data from the pH 7 samples can be fit with two U–U shells at 3.87 and 4.61 Å. This U(VI) coordination environment is consistent with that of metaschoepite (Table S4) as the dominant crystalline component in both of the samples. In contrast, the U(VI) local environment from the pH 11 samples has an additional U–O shell at 2.86 Å, and U–U shells at 3.76 ad 3.94 Å. These parameters are most similar to those for U(VI) in Na-compreignacite (Table S4). The analysis of the EXAFS and XANES data shows the consistency between the use of the two techniques (XAS and luminescence spectroscopy), in terms of the key U(VI) phases in the HL system with metaschoepite as the dominant solid phase at pH 5.5 and 7, and Na-compreignacite forming at pH 11. Note that it was not possible to resolve the contribution of adsorbed U(VI) to the EXAFS spectra, likely due to the lack of long-range order for these species. However, these results highlight the advantage of luminesence over XAS which allows for a greater accuracy in term of phase/species identifcation and quantification for U(VI) in complex systems.

The discrepancy observed between the geochemical model (Figure 1) and the experimental data for the LL experiment (Figure 4) highlights the impact the lack of available thermodynamic data for the sorption of uranyl carbonate species on gibbsite has on the ability of the geochemical model to reproduce the results observed experimentally. Additions to the PHREEQC model are therefore required to better represent the experimental data; in lieu of thermodynamic studies, exploratory modifications were carried out. The geochemical model for the low level system was altered by suppressing the formation of the uranyl(VI) carbonate solid phase and adding values for sorption of uranyl(VI) to the gibbsite surface via coordination of the aqueous UO_2_(CO_3_)_3_^4–^ species (the dominant species in solution) to the gibbsite surface as ≡AlO(UO_2_)(CO_3_)_2_^3–^, to obtain a model which more accurately represents the removal of uranyl(VI) from solution. The log K value required for the model to predict full removal of U(VI) from solution (≥97%) is 15 (Table S2), which is much higher than experimentally observed log K values for the sorption of uranyl(VI) carbonate on orthoclase (8.96), muscovite (8.71) and kaolinite (9.9)^45,46^ but nevertheless highlights the necessity for further experimental understanding for the development of representative geochemical models. While outside the scope of this work, further analyses could be used alongside these results to yield the boundary concentrations for the formation of precipitates.

In summary, four types of uranyl species have been identified in the U(VI)-gibbsite system through the combination of time-resolved and steady luminescence spectroscopy on samples from two experimental U(VI) loadings. Two sorption complexes are the only luminescent species present at 25 μM U(VI) in solution (LL experiments), a concentration below the precipitation limit of uranium phases, at the pH range studied, as indicated by PHREEQC geochemical modeling. A uranyl hydroxide bidentate sorption species, ≡AlO_2_–UO_2_(OH), is formed below pH 8, a second sorption carbonate-containing bidentate complex, ≡AlO_2_–UO_2_(CO_3_)_2_^4–^, is present throughout the experimental pH range. In the higher concentration regime (HL experiments), two precipitates, metaschoepite and Na-compreignacite form alongside these sorption complexes. Metaschoepite is the main contributor to the total luminescence intensity between pH 5 and 9, after which point Na-compreignacite and the sorption complexes become more prevalent and their formation more thermodynamically favorable.

Together the data presented herein demonstrate the ability of gibbsite to adsorb uranyl(VI) carbonate species from solution at alkaline as well as circumneutral pH values. Furthermore, the analytical approach shows the application of luminescence as a technique to identify and quantify a wide range of uranyl(VI) species (e.g., solids and adsorbed complexes) in complex multiphase systems, particularly when large data sets are combined with appropriate deconvolution methods. Further quantification of this system could be carried out by using the obtained species and sorption studies to derive a thermodynamic model for the sorption associated with other mineral phases and more complex environmental samples (e.g., soils) containing multiple phases. These techniques (i.e., luminescence, XAS and geochemical modeling) provide a powerful toolbox for the interpretation of emission fingerprints and hence robust assignments of emissive uranyl(VI) species associated with complex environmental samples containing multiple components. This is paramount to elucidating geochemical sorption of key radionuclides and moving forward will contribute to a better understanding of actinide environmental mobility and retention processes central to the study of contaminated environments and the disposal of radioactive wastes.

## Supporting Information Available

Additional information on the solid phase synthesis, luminescence analysis, XAS analysis and geochemical modeling (PDF)

## Abbreviations

EEM: excitation emission matrix
EXAFS: extended X-ray absorption fine structure
HL: high loading
LL: low loading
PARAFAC: parallel factor analysis
TRES: time-resolved emission spectrum
TRLFS: time-resolved laser-induced fluorescence spectroscopy
XAS: X-ray absorption spectroscopy
