# Supplementary material for: Identification and Quantification of Multiphase U(VI) Speciation on Gibbsite with pH Using TRLFS and PARAFAC of Excitation Emission Matrices
Source: Environ Sci Technol. 2024 Sep 24;58(40):17916–25. doi: 10.1021/acs.est.4c06133 (PMC11466309; doi:10.1021/acs.est.4c06133)
Supplement: Supplementary file 1 — es4c06133_si_001.pdf [file es4c06133_si_001.pdf]

Supplementary Information

Identification and Quantification of Multiphase U(VI) Speciation on Gibbsite with pH using TRLFS and PARAFAC of Excitation Emission Matrices

*Laura Lopez-Odriozola,[1] Samuel Shaw,[2] Liam Abrahamsen-Mills,[3] Charlotte Waters,[2] Louise S. Natrajan[1]**

[1] Centre for Radiochemistry Research, Department of Chemistry, The University of Manchester, Manchester, M13 9PL, U.K.

[2] Research Centre for Radwaste Disposal and Williamson Research Centre for Molecular Environmental Science, Department of Earth and Environmental Sciences, The University of Manchester, Manchester, M13 9PL, U.K.

[2] National Nuclear Laboratory, Warrington, WA3 6AE, Cheshire, U.K.

Summary: 26 pages, 25 Figures including 13 Graphical Figures, 4 Tables

This document provides supporting information for this paper, including details of syntheses of solid phases, geochemical modelling, luminescence data, parallel factor analysis (PARFAC), species assignments and XAS analysis and fitting

Contents

Section S1: Solid Phase Synthesis S3
Section S2: Sorption and PHREEQC Geochemical Modelling S6
Section S3: Luminescence data S9
Section S4: Parallel Factor Analysis (PARAFAC) S13
Section S5: Species Assignments S17
Section S6: X-Ray Absorption Spectroscopy analysis S22
REFERENCES S26

.

Section S1: **Solid Phase Synthesis**

Gibbsite

$Al(NO_3)_3{\cdot}9H_2O$ (75.2 g, 0.2 mol) was dissolved in 800 mL of MilliQ deionised (DI) water, the pH adjusted to 5 with a 5 M NaOH solution and mixture stirred for one hour at room temperature. This was centrifuged at 8000 rpm for 20 minutes to collect a gel. After three washes with DI water, the gel was dispersed in 400 mL of DI water to make a 0.5 M Al^{3+} solution and the pH was adjusted to 5 with 5 M NaOH. The suspension was left in an oven at 80 °C for 160 hours and shaken periodically. Gibbsite (14 g, 0.18 mol, 90% yield) was synthesised and characterised using powder X-ray diffraction (PXRD). The surface area of the gibbsite was determined to be 32.89 ± 0.03 m^2/g using N_2-BET (Brunauer–Emmett–Teller), and the PZC (point of zero charge) of the gibbsite was determined to be 8.6 ± 0.5 by zeta potential measurements.

Metaschoepite ($(UO_2)_8O_2(OH)_{12} \bullet 10H_2O$)

$UO_2(NO_3)_2.6H_2O$ (1.181 g, 2.35 mmol) was dissolved in 235 mL of MilliQ DI water to make a 0.01 M U(VI) solution. The pH was adjusted to 5.9 with 0.5 M NaOH and left stirring for 10 days. The precipitate was isolated by centrifugation, washed with DI water three times and dried at 40 °C for 24 hours. The metaschoepite (0.73 g, 2.27 mmol, 97 % yield) was characterised using PXRD.

Na-Compreignacite ($Na_2(UO_2)_6O_4(OH)_6{\bullet}7H_2O$):

The synthesised metaschoepite (0.286 g, 0.89 mmol) and 0.09 g Na_2CO_3 were dissolved in 5 mL of MilliQ DI water, the pH was adjusted to 6 using 0.2 M NaOH. The solution was sealed and heated at 80 °C with constant shaking for 4 days. The precipitate was separated by centrifugation, washed with Na_2CO_3 solution three times and dried at 40 °C for 48 hours. The Na-compreignacite

(0.185 g) was identified to have the same structure as the sodium analogue of K-compreignacite[1] by PXRD.

$K_3Na(UO_2)(CO_3)_3.H_2O$

A synthetic potassium-sodium mineral with uranyl tricarbonate clusters, analogous to $Na_4(UO_2)(CO_3)_3$, was synthesised according to the literature procedure.[2] $UO_2(NO_3)_2.6H_2O$ (0.508 g, 1.58 mmol) was dissolved in 10 mL DI water, KNO_3 (0.6 g, 5.93 mmol) was added, followed by Na_2CO_3 (0.3 g, 2.83 mmol) and stirred vigorously for two minutes. The solution was left undisturbed at room temperature for seven days. After the water evaporated, the subsequent solid was washed three times (2 x IPA, 1 x ethanol) and dried at room temperature to a constant weight. The solid was characterised by PXRD.

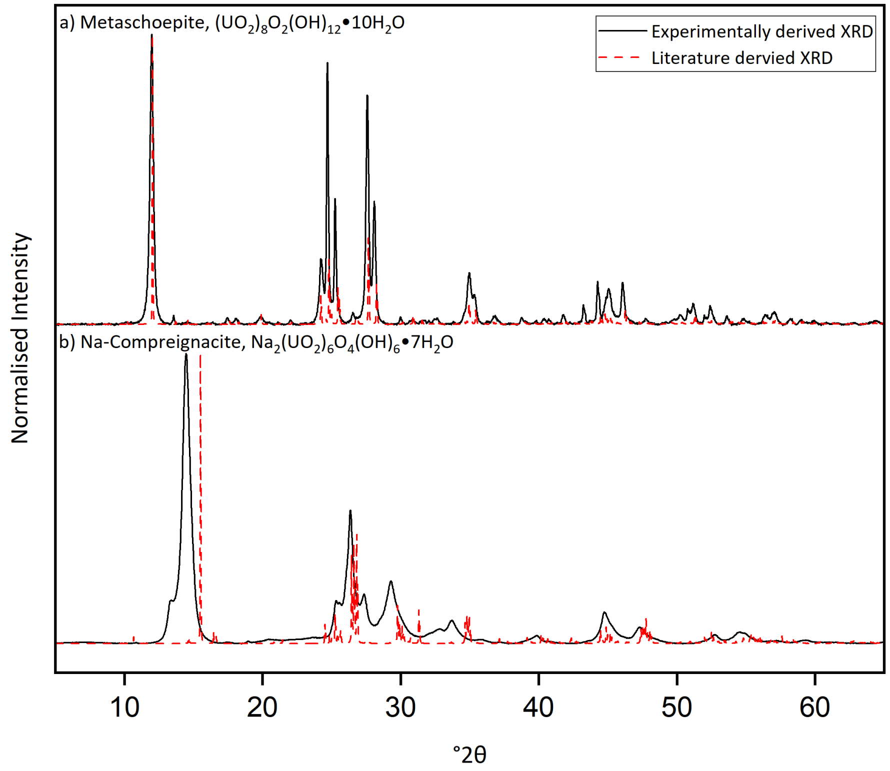

Figure S1. Metaschoepite and Na-Compreignacite XRD experimentally derived XRD patterns and literature derived XRD patterns. As Na-Compreigancite is analogous to K-compreignaicte, the literature XRD of the K-analogue is shown. Literature XRD has been derived from CIFs.[1,3]

Section S2: **Sorption and PHREEQC Geochemical Modelling**

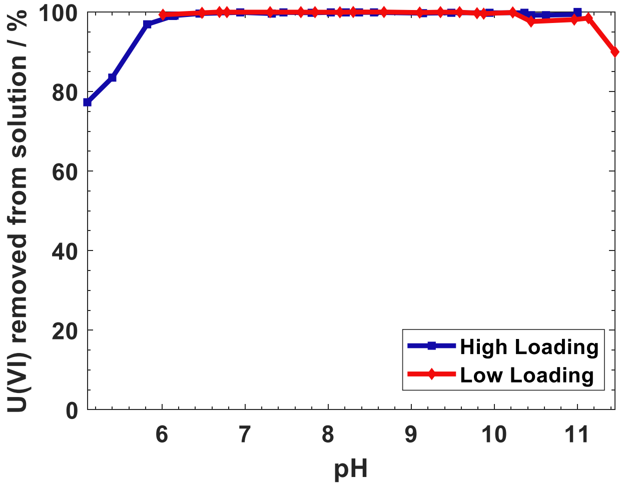

Figure S2. Graph depicting the U(VI) removed from solution at each pH for the high loading and low loading experiments determined by colorimetery using Bromo-PADAP and ICP-MS respectively.

Table S1. Relevant reactions and corresponding log K values extracted from Karamalidis and Dzombak.[4]

Reaction	log K
$\equiv AlOH + H^+ = \equiv AlOH_2^+$	7.17
$\equiv AlOH = \equiv AlO\text{-} + H^+$	-11.18
$\equiv AlOH + UO_2^{2+} = \equiv AlOUO_2^+ + H^+$	1.22
$\equiv AlOH + UO_2^{2+} + 3H_2O = \equiv AlOUO_2(OH)_3^{2-} + 4H^+$	-22.0
$\equiv AlOH + 3UO_2^{2+} + 5H_2O = \equiv AlO(UO_2)_3(OH)_5 + 6H^+$	-15.6
$\equiv AlOH + CO_3^{2-} = \equiv AlHCO_3 + H_2O$	21.48
$\equiv AlOH + CO_3^{2-} = \equiv AlCO_3^- + H_2O$	15.93

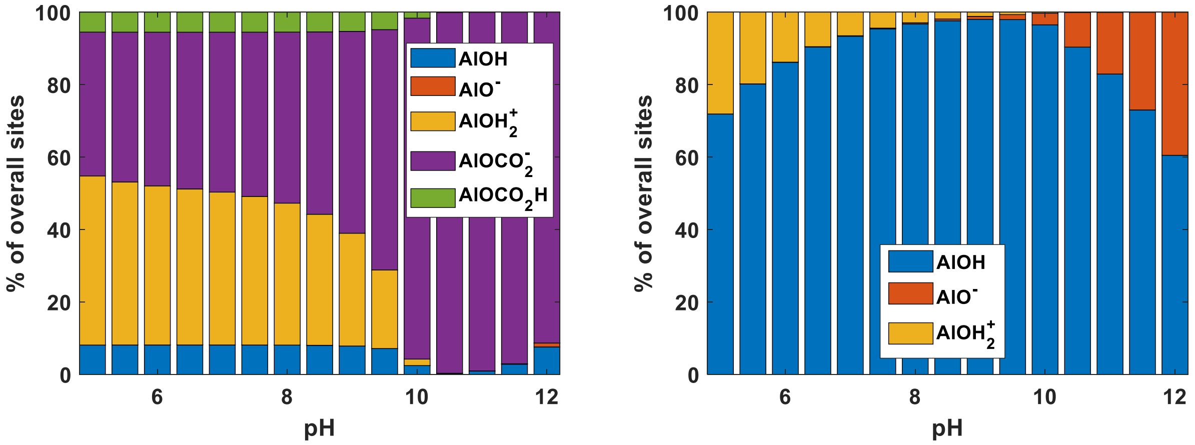

Figure S3. Gibbsite surface site speciation predicted by PHREEQC with the inclusion (left) and exclusion (right) of carbonate complexation constants

Table S2. Percentage of U(VI) in solution and sorbed to gibbsite from exploratory PHREEQC modelling at each pH on changing the trial log K value for the proposed sorption of a uranyl carbonate to the gibbsite surface, from an initial concentration of 25 µM U(VI). The table is colour coded based on agreement with experimental observations for ease of interpretation, green indicates good agreement (>97%), orange poor agreement (>65%), red disagreement (<65%).

pH	log K = 9		log K = 12		log K = 13		log K = 14		log K = 15	
	Solution (%)	Sorbed (%)	Solution (%)	Sorbed (%)	Solution (%)	Sorbed (%)	Solution (%)	Sorbed (%)	Solution (%)	Sorbed (%)
5	2	98	0	100	0	100	0	100	0	100
5.5	0	100	0	100	0	100	0	100	0	100
6	0	100	0	100	0	100	0	100	0	100
6.5	0	100	0	100	0	100	0	100	0	100
7	0	100	0	100	0	100	0	100	0	100
7.5	0	100	0	100	0	100	0	100	0	100
8	1	99	0	100	0	100	0	100	0	100
8.5	35	65	0	100	0	100	0	100	0	100
9	98	3	4	96	0	100	0	100	0	100
9.5	100	0	71	29	20	80	3	97	0	100
10	100	0	97	3	75	25	24	76	3	97
10.5	100	0	25	75	3	97	0	100	0	100
11	97	3	3	97	0	100	0	100	0	100

Section S3: **Luminescence data**

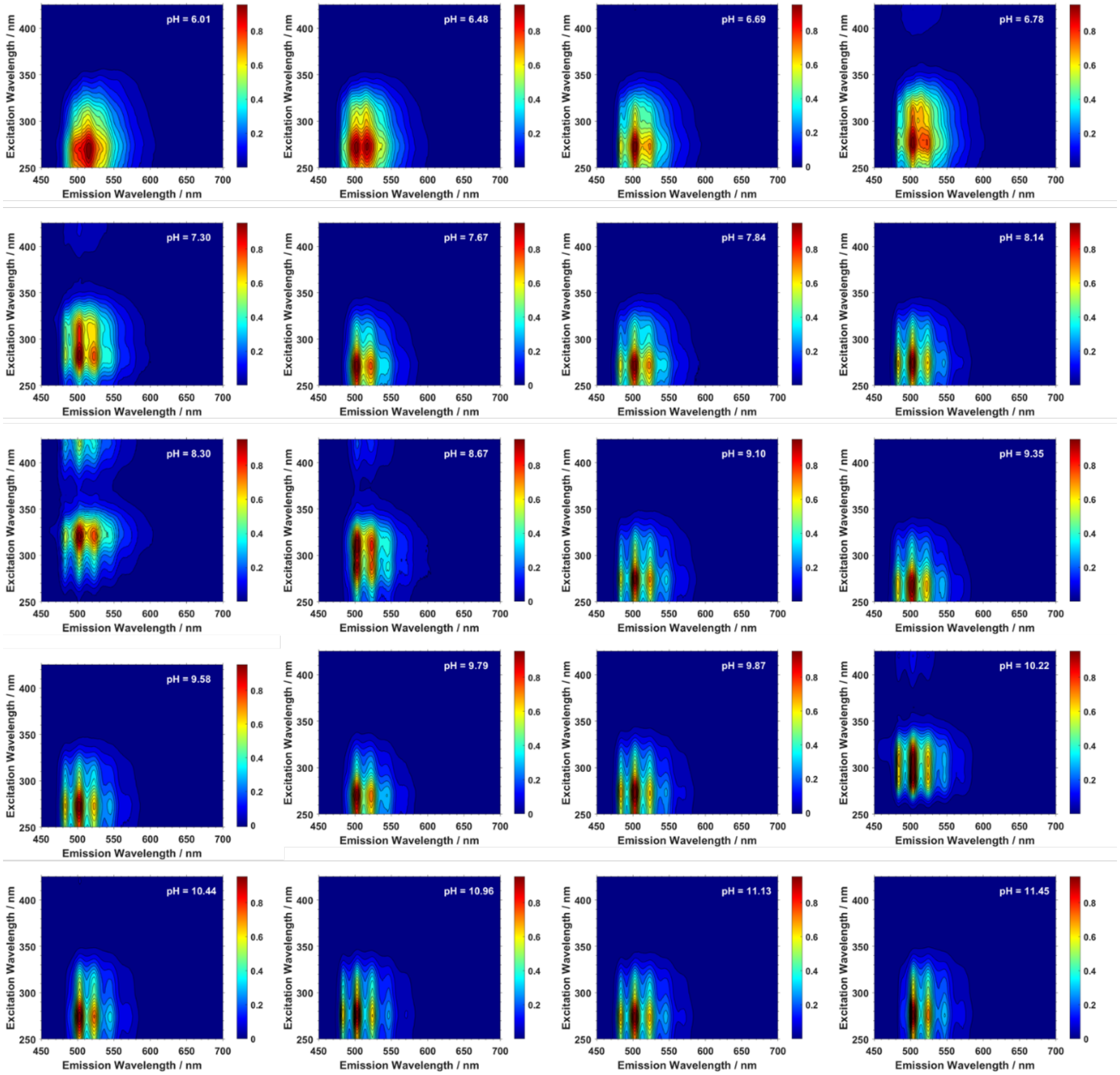

Figure S4. Excitation emission matrices (EEMs) for the low loading (LL) samples, in order of ascending pH. Recorded at 20 K with emission wavelengths between 450 and 700 nm for incident excitation of 250 – 430 nm in 5 nm steps.

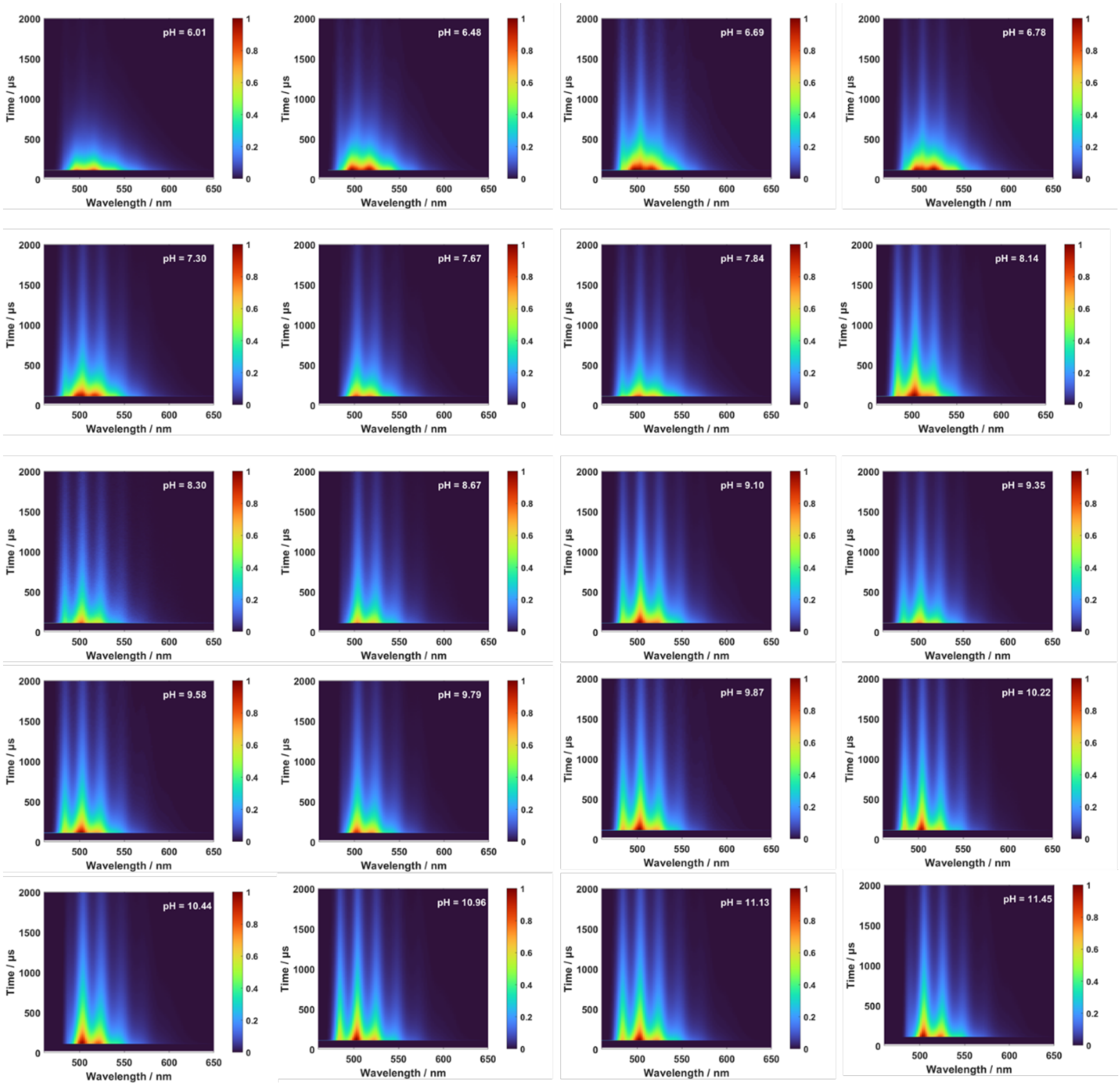

Figure S5. Time-resolved emission spectra (TRES) for the low loading (LL) samples, in order of ascending pH, recorded at 20 K following 285 nm excitation.

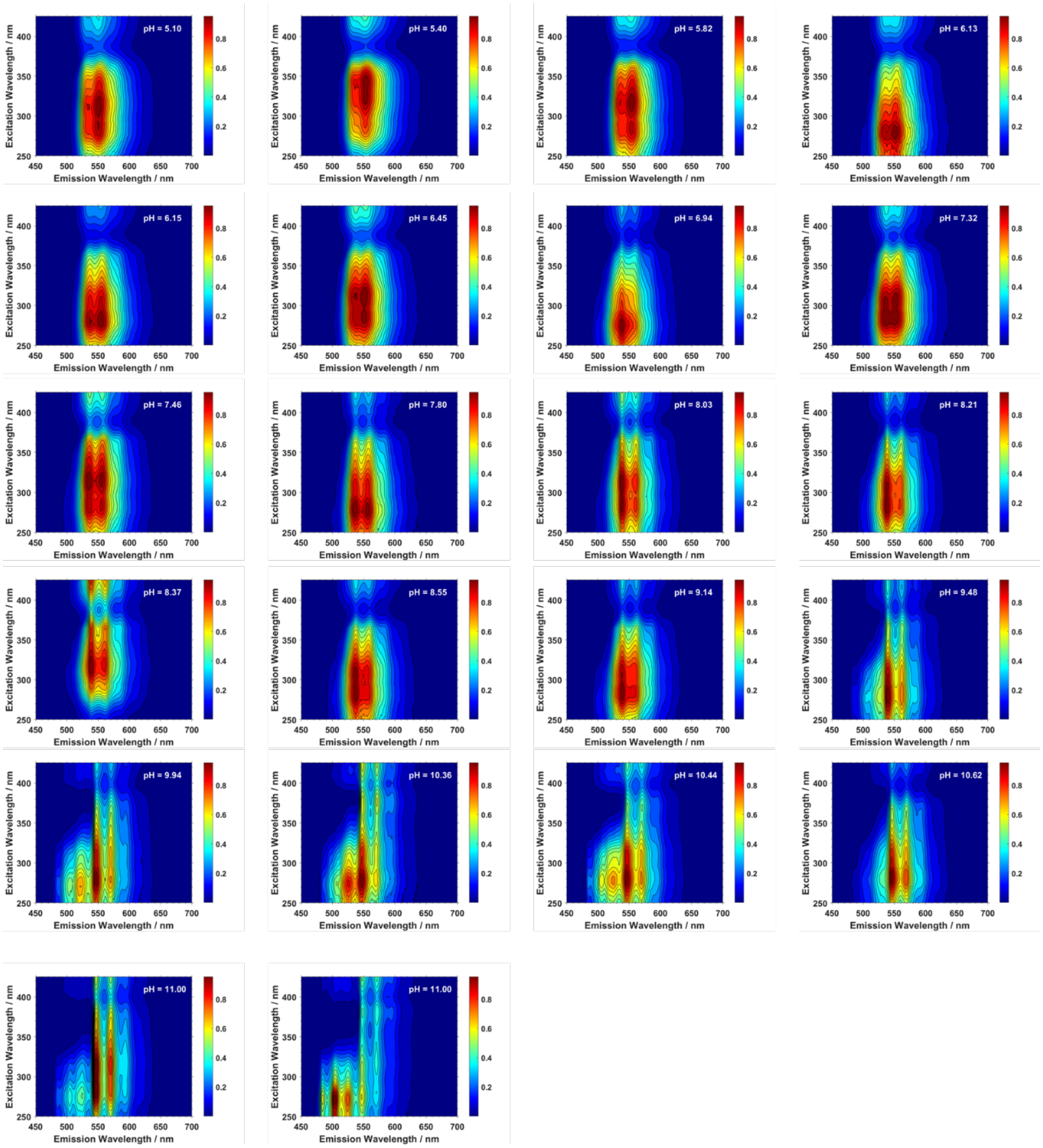

Figure S6. Excitation emission matrices (EEMs) for the high loading (HL) samples, in order of ascending pH. Recorded at 20 K with emission wavelengths between 450 and 700 nm for incident excitation of 250 – 430 nm in 5 nm steps.

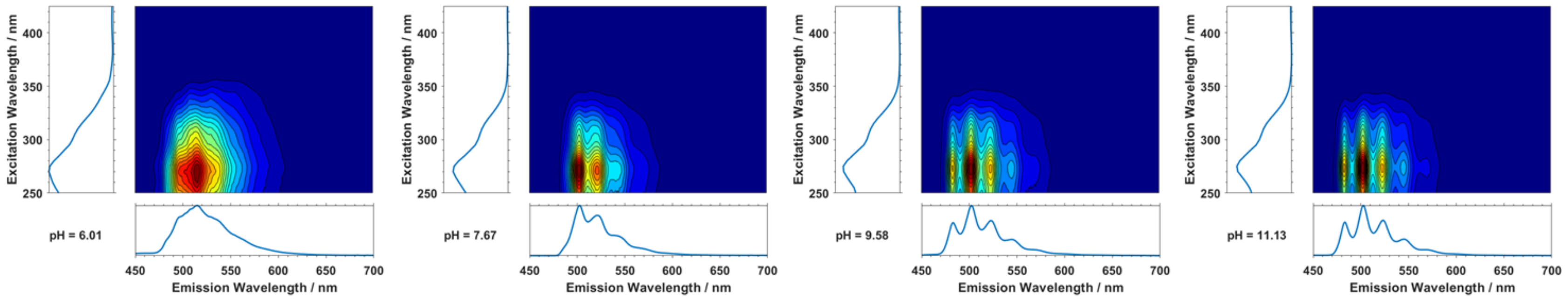

Figure S7. Normalised excitation emission maps with emission and excitation traces of selected low loading samples at pH (L-R) 6.0, 7.7, 9.6 and 11.1 recorded at 20 K, showing the constant excitation profile throughout the experimental range.

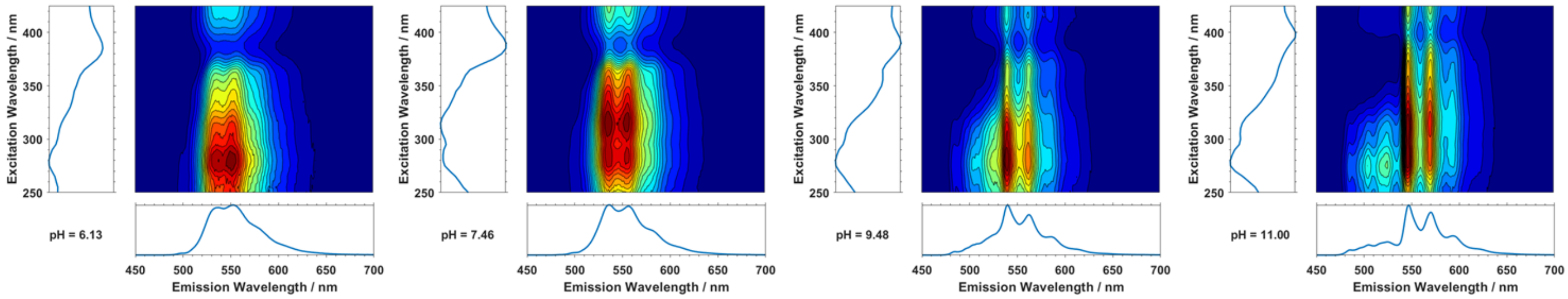

Figure S8. Normalised excitation emission maps with emission and excitation traces of selected high loading (HL) samples at pH (L-R) 6.1, 7.5, 9.5 and 11.0 recorded at 20 K, showing the lack of consistency in the excitation profile throughout the experimental range.

Section S4: **Parallel Factor Analysis (PARAFAC)**

The 3 dimensional dataset for PARAFAC deconvolution was generated by compiling the EEMs from each HL sample. In the exploratory phase of the modelling, the excitation wavelength was cropped to include only values lower than 395 nm and two outlier samples (pH 8.37 and pH 11.00) were removed from the dataset after the initial Jack-Knife analysis. The low intensity of emission between 395 nm and 405 nm excitation results in a significant number of zero and near zero values in the dataset, which impact the ability of PARAFAC to model the data.[5] Additionally, excitations at > 395 nm (directly into the UO_2^{2+} ligand to metal charge transfer electronic transition region) have been reported to be site selective, resulting in a loss of trilinearity in this case.[6]

Both the sum of square residuals (SSR) and the variance explained exhibit an elbow in the curve at 3 components, thus additional components do not provide significant improvements to the model, furthermore the core consistency analysis also suggests 3 components are most representative of the sample.

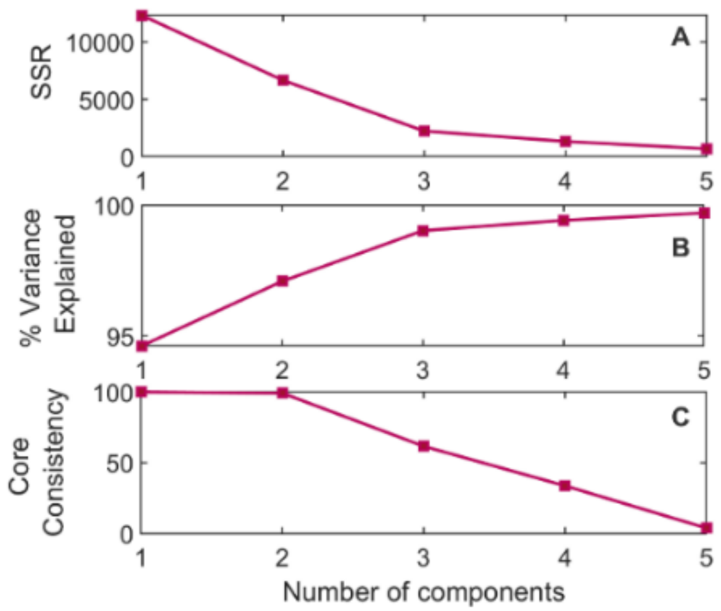

Figure S9. (*A)* Sum of square residuals (SSR), (B) percentage of variance explained and core consistency (C) for PARAFAC models of 1 – 5 components, showing the data are best represented by 3 components.

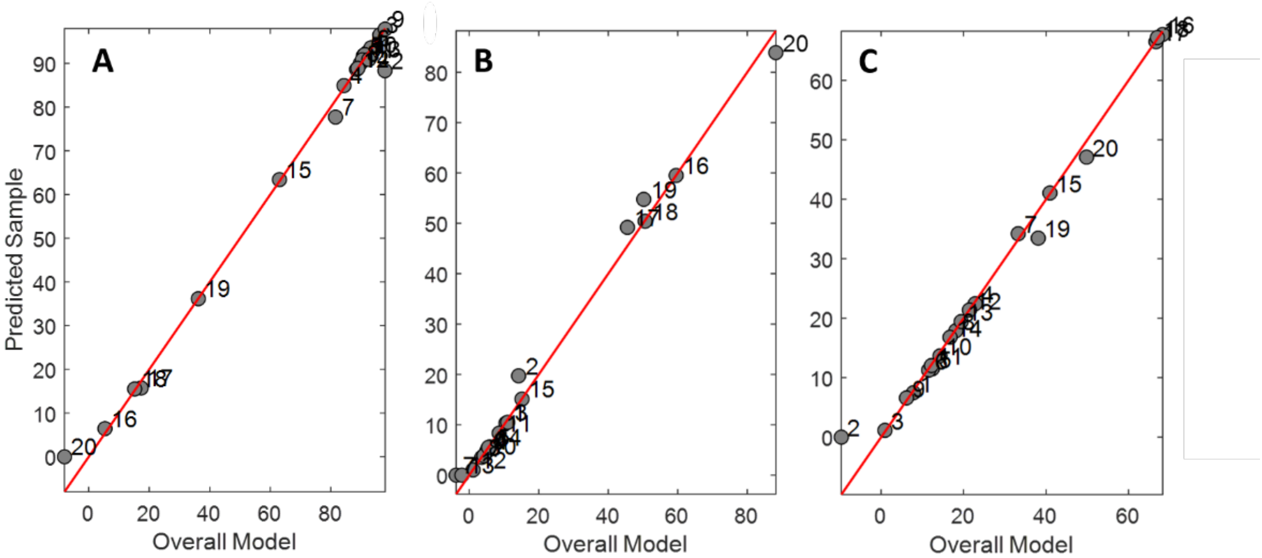

Figure S10. Final Jack-knife analysis for the final model for (A) component 1, (B) component 2 and (C) component 3.

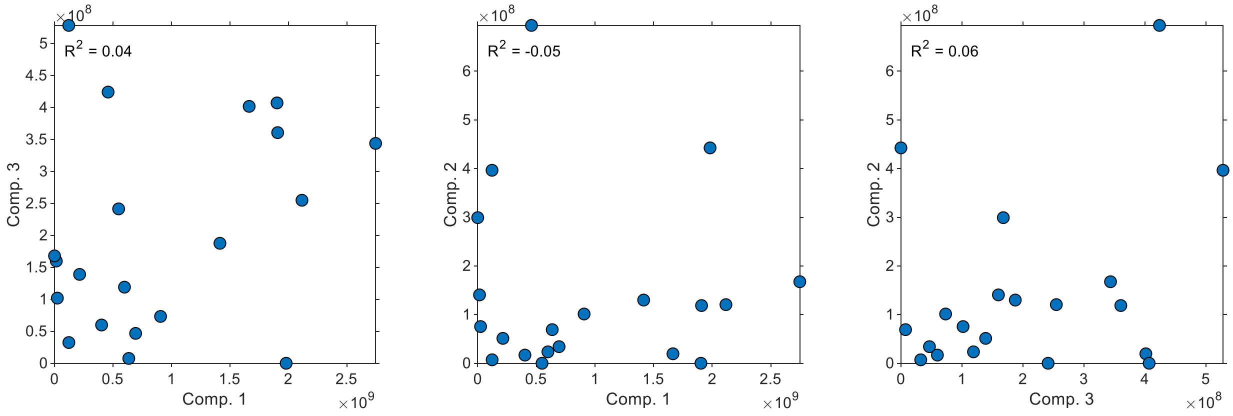

Figure S11. Component correlation plots for the components, demonstrating each component represents a unique species.

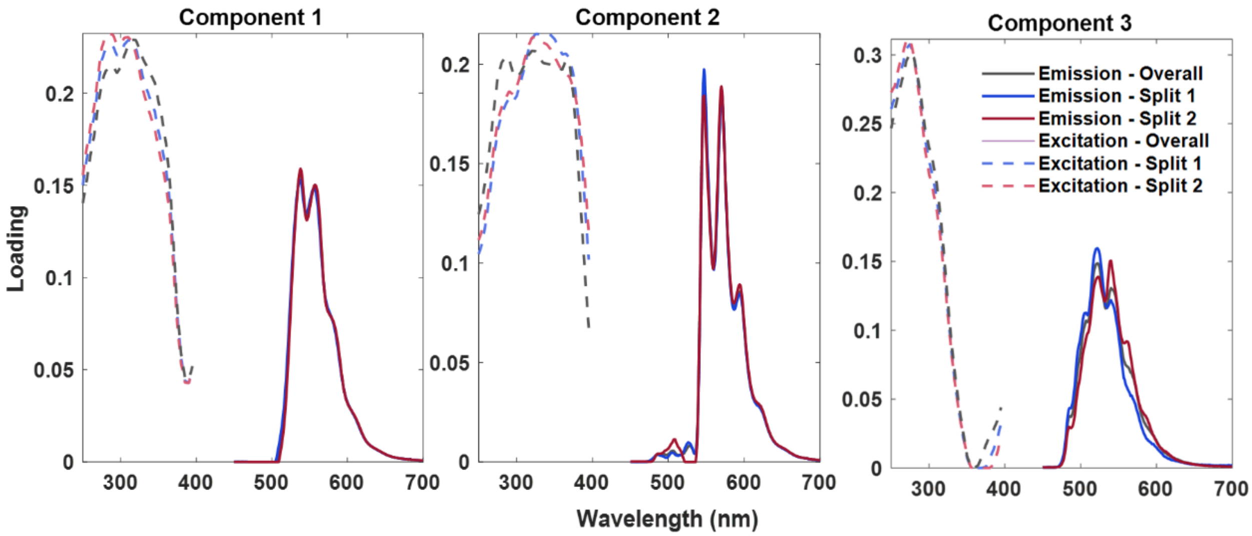

Figure S12. Split half validation for (left-right) components 1, 2 and 3, in the final model.

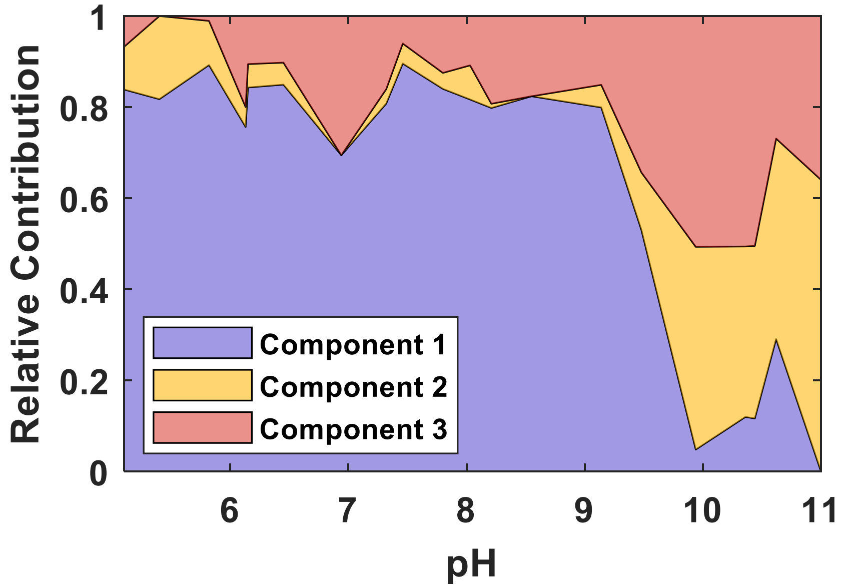

Figure S13. Area graph of the contribution to the emission intensity from each of the components obtained from PARAFAC deconvolution.

Section S5: **Species Assignments**

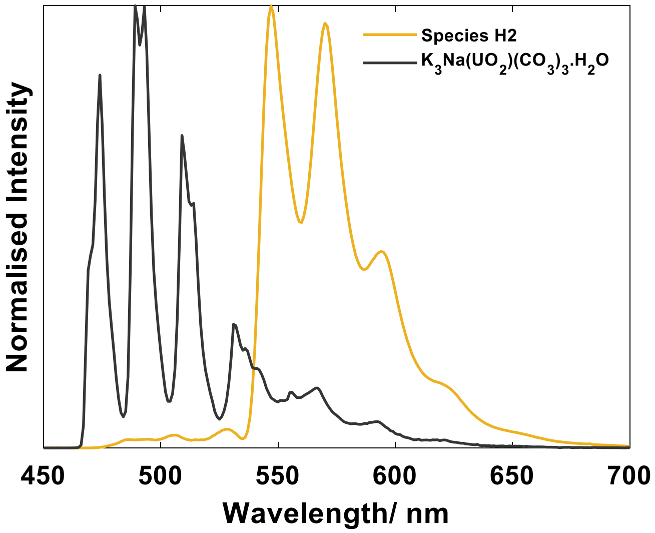

Figure S14. Comparison of the emission spectrum of H2 determined from PARAFAC and that of a synthesised mineral analogous to $Na_4(UO_2)(CO_3)_3$, at 20 K following 285 nm excitation.

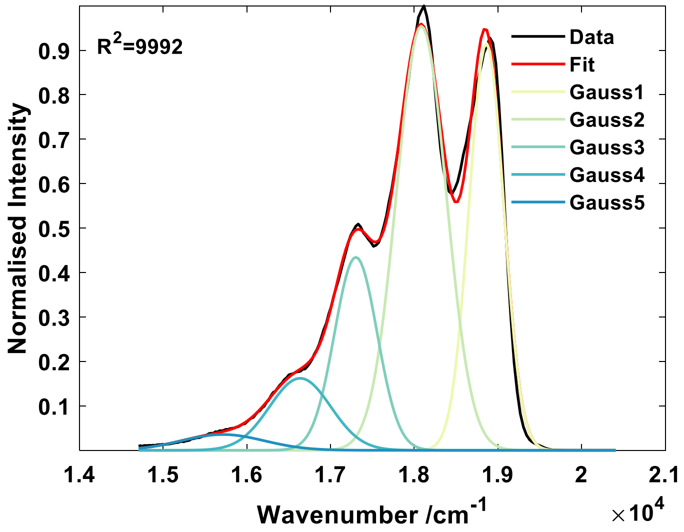

Figure S15. Gaussian deconvolution for the emission spectrum of the metaschoepite standard following 285 nm excitation at 20 K

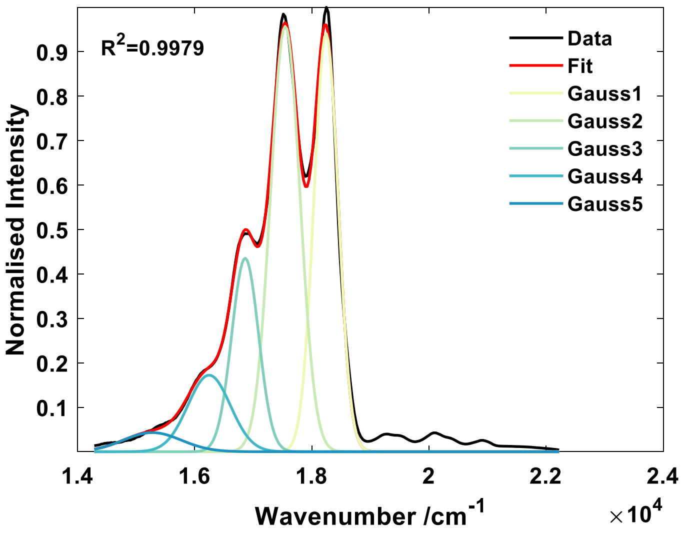

Figure S16. Gaussian deconvolution for the emission spectrum of the Na-compreignacite standard following 285 nm excitation at 20 K

.

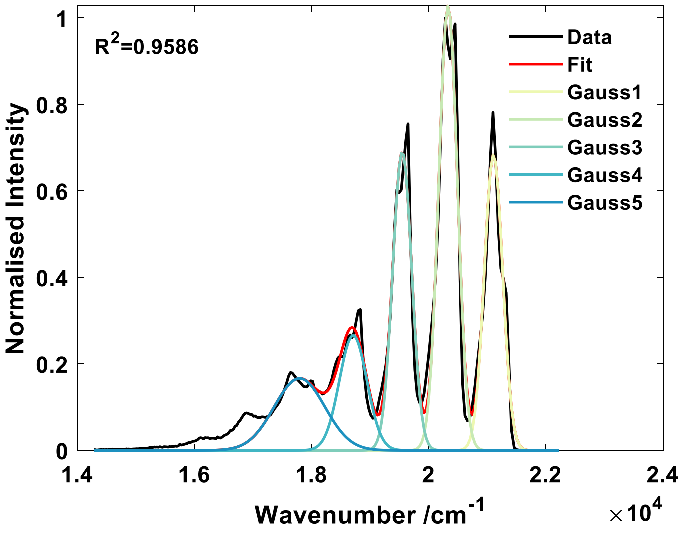

Figure S17. Gaussian deconvolution for the emission spectrum of the $K_3Na(UO_2)(CO_3)_3.H_2O$ standard following 285 nm excitation at 20 K

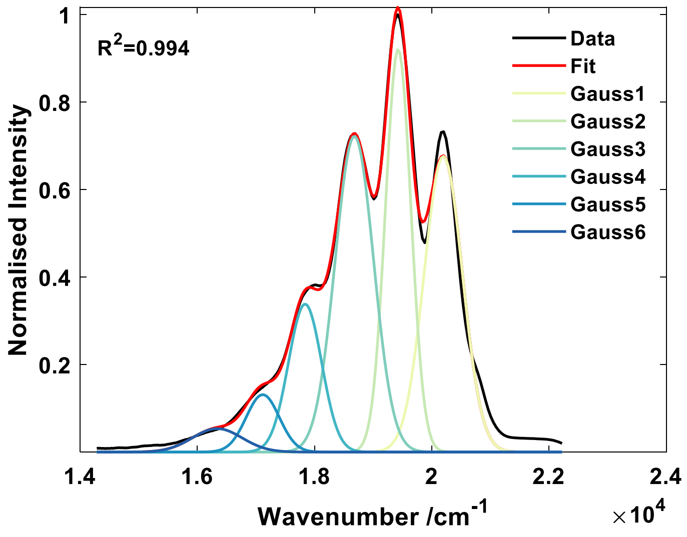

.

Figure S18. Gaussian deconvolution for the emission spectrum discerned for L1 from time resolved emission spectroscopy following excitation at 285 nm.

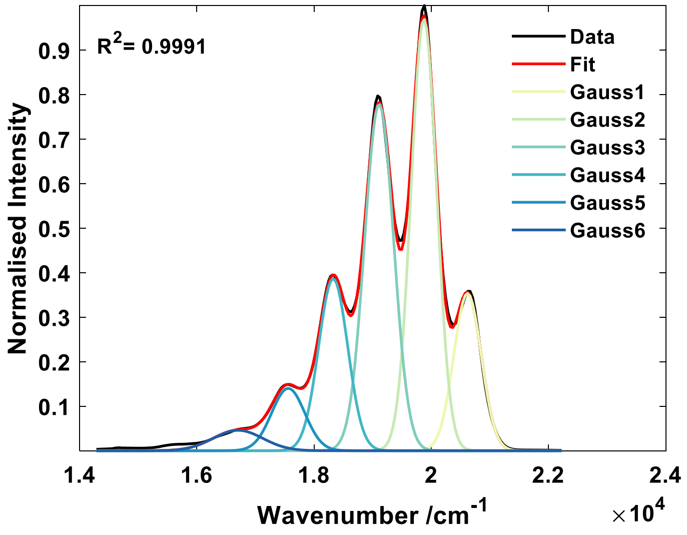

Figure S19. Gaussian deconvolution for the emission spectrum discerned for L2 from time resolved emission spectroscopy following excitation at 285 nm.

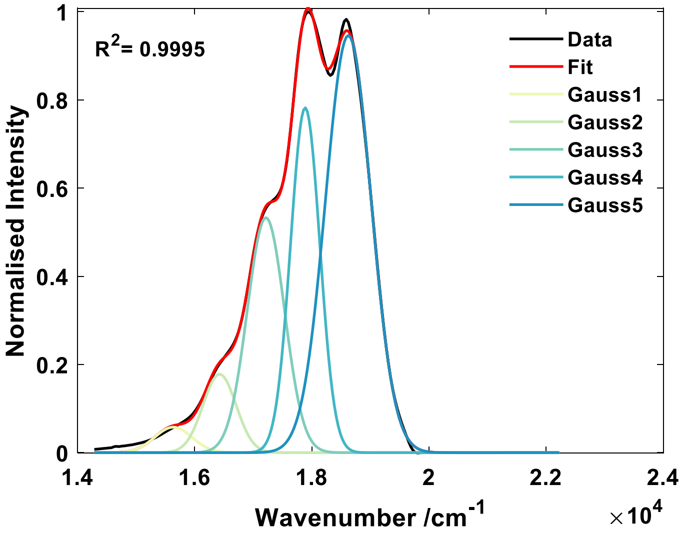

Figure S20. Gaussian deconvolution for the emission spectrum calculated for H1 from PARAFAC on EEMs of the HL sorption set.

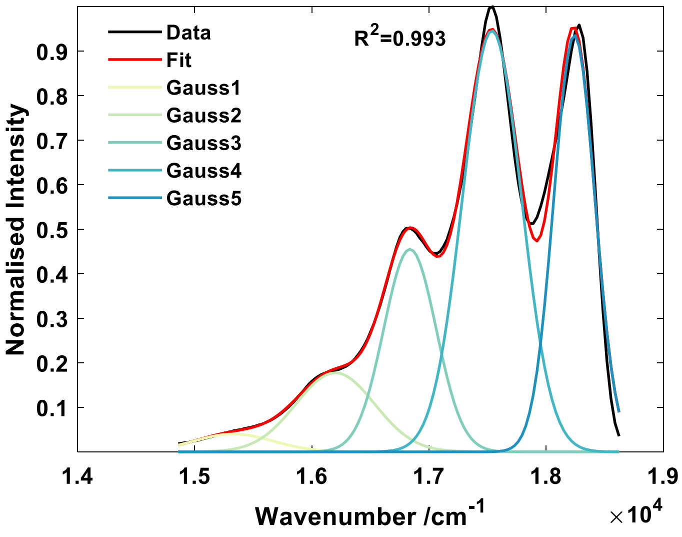

Figure S21. Gaussian deconvolution for the emission spectrum calculated for H2 from PARAFAC on EEMs of the HL sorption set.

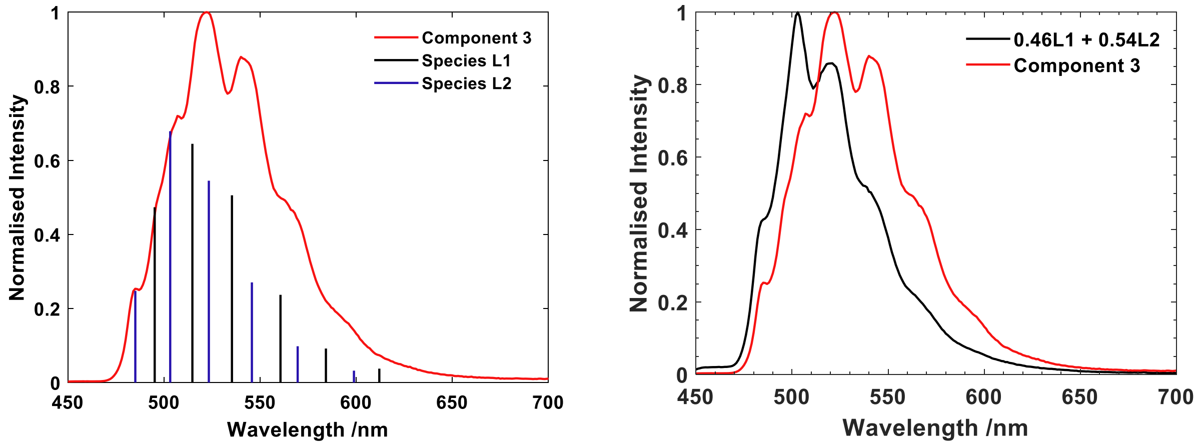

Figure S22. Superposition of peaks of species L1 and L2 and component 3 spectrum (left), and sum of the two species to show the most representative ratio (right). Whilst the peak positions are in the same place, the ratios (heights) are different. As the ratio of species A and B contributing to component 3 throughout the pH range is likely to be inconsistent, component 3 is likely to be a weighted average of the contributions of species A and B throughout the pH range and thus more complex than a 'true' sum of the two spectra.

Section S6: **X-Ray Absorption Spectroscopy analysis**

Table S3. Linear combination fitting (LCF) of the three experimental samples as combinations of the two minerals from their L_3-edge XANES data.

**Sample**	Metaschoepite (%)	Na-compreignacite (%)	R-factor
**HL pH 5.5**	80.9	19.1	0.000805
**HL pH 7**	96.7	4.8	0.000338
**HL pH 11**	29.5	70.5	0.0002

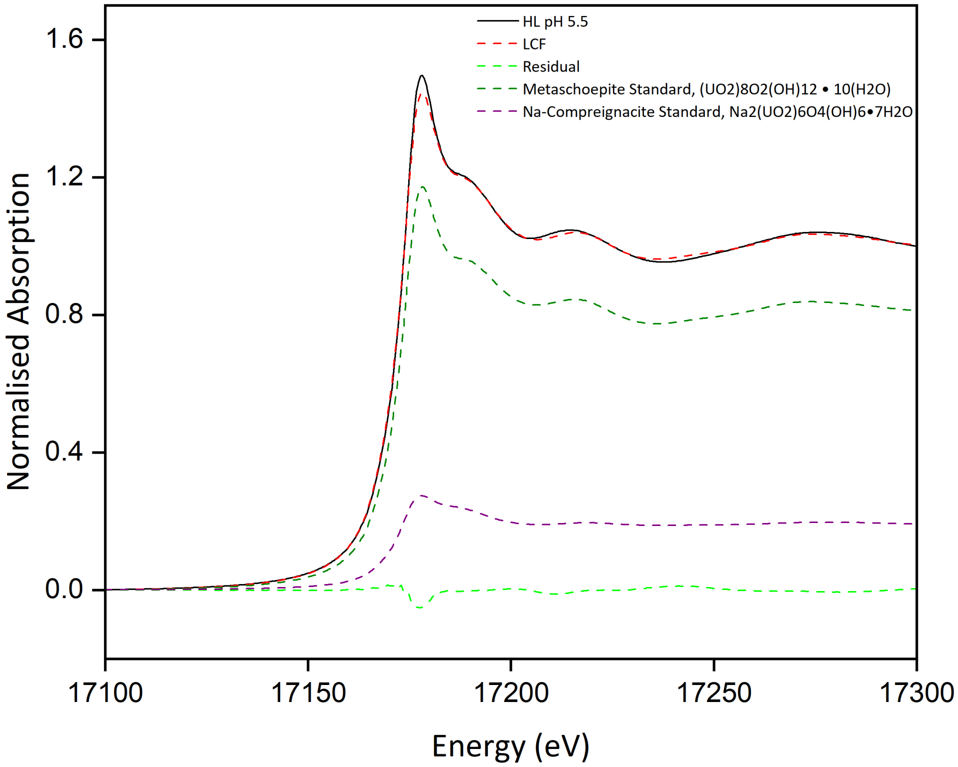

Figure S23. Linear combination fit (LCF) of the L_3-edge XANES of the HL pH 5.5 sample to the metaschoepite and Na-compreignacite standards.

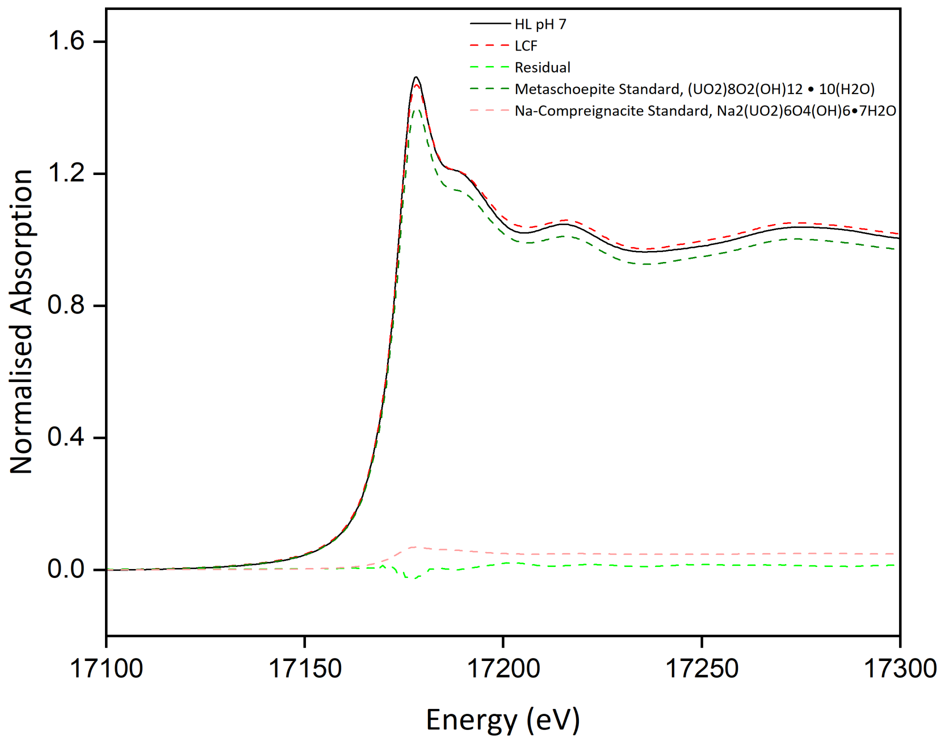

Figure S24. LCF of the L_3-edge XANES of the HL pH 7.0 sample to the metaschoepite and Na-compreignacite standards.

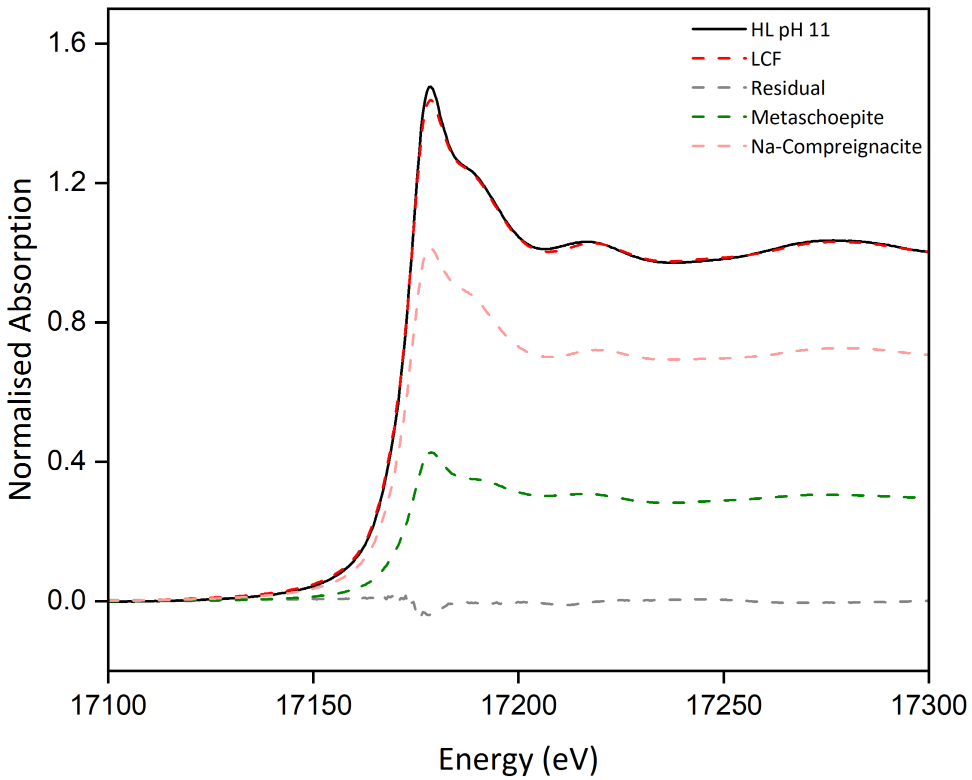

Figure S25. LCF of the L_3-edge XANES of the HL pH 11.0 sample to the metaschoepite and Na-compreignacite standards.

Table S4. EXAFS best-fit pitting parameters for the HL samples at pH 5.5, 7.0 and 11.0, and the two mineral standards. Including coordination number (CN), U interatomic distances (R), Debye-Waller factors (σ^2), amplitude factor (S_0^2), shift in energy from calculated fermi level (ΔE_0), and goodness of fit (R-factor). Multiple scatterers are denoted by MS and are defined using parameters derived for single scattering paths. The fitting ranges for all samples, in both k and R are also shown.

Sample	Path	CN	R(Å)	σ^2(Å^2)	S_0^2	ΔE_0 (eV)	R-Factor	Fitting Range k	Fitting Range R
HL pH 5.5	U-O_{ax}	2	1.80	0.00169 (8)	0.882	11 (3)	0.0179	3-13.3	1.15 – 2.8
	U-O_{eq}	2.6	2.26	0.006 (5)					
	U-O_{eq}	3.4	2.42	0.005 (4)					
HL pH 7.0	U-O_{ax}	2	1.80	0.0033(3)	1.1	9.9 (7)	0.0057	3 – 13.4	1.5 - 5
	U-O_{eq}	2	2.25	0.006 (1)					
	U-O_{eq}	2	2.40	0.006 (1)					
	U-U	1.2	3.87	0.0033 (8)					
	U-U	1.5	4.61	0.007 (3)					
	U-O MS rattle	2	3.61	0.0067 (3)					
	U-O MS	2	3.61	0.0067 (3)					
HL pH 11.0	U-O_{ax}	2	1.82	0.0028 (5)	1	11 (2)	0.0149	3 - 13.3	1-4.3
	U-O_{eq}	2	2.27	0.003 (1)					
	U-O_{eq}	2	2.42	0.003 (2)					
	U-O_{eq}	1.3	2.86	0.0037 (3)					
	U-U	2	3.76	0.006 (3)					
	U-U	1.2	3.94	0.0028 (2)					
Metaschoepite Standard	U-O_{ax}	2	1.801 (5)	0.0026 (4)		12(1)	0.0209	3 – 13.7	1.15 - 5

	U-O_{eq}	1	2.21 (1)	0.003 (2)				
	U-O_{eq}	3.4	2.39 (1)	0.009 (2)				
	U-U	1.2	3.87 (1)	0.002 (1)				
	U-U	1.5	4.55 (3)	0.006 (3)				
	U-O MS	2	3.602 (5)	0.0052 (4)				
	U-O Rattle	2	3.602 (5)	0.0052 (4)				
Na-Compreignacite	U-O_{ax}	2	1.83	0.005 (1)	6 (1)	0.0205	3 – 11.8	1.2 – 4.5
	U-O_{eq}	3	2.22	0.004 (1)				
	U-O_{eq}	2	2.87	0.008 (4)				
	U-U	1.3	3.71	0.004 (2)				
	U-U	1.2	4.21	0.10(1)				
	U-O MS	2	3.6	0.0067 (1)				

REFERENCES

(1) Li, Y.; Burns, P. C. The Structures of Two Sodium Uranyl Compounds Relevant to Nuclear Waste Disposal. *J. Nucl. Mater.* **2001**, *299* (3), 219–226. https://doi.org/10.1016/S0022-3115(01)00702-4.

(2) Li, Y.; Burns, P. C. THE CRYSTAL STRUCTURE OF SYNTHETIC GRIMSELITE, K3Na[(UO2)(CO3)3](H2O). *Can. Mineral.* **2001**, *39* (4), 1147–1151. https://doi.org/10.2113/gscanmin.39.4.1147.

(3) Weller, M. T.; Light, M. E.; Gelbrich, T. Structure of Uranium(VI) Oxide Dihydrate, $UO_3 \cdot 2H_2O$; Synthetic *Meta* -Schoepite $(UO_2)_4O(OH)_6 \cdot 5H_2O$. *Acta Crystallogr. Sect. B Struct. Sci.* **2000**, *56* (4), 577–583. https://doi.org/10.1107/S0108768199016559.

(4) Karamalidis, A. K.; Dzombak, D. A. *Surface Complexation Modeling*; John Wiley & Sons, Inc.: Hoboken, NJ, USA, 2010. https://doi.org/10.1002/9780470642665.

(5) Andersen, C. M.; Bro, R. Practical Aspects of PARAFAC Modeling of Fluorescence Excitation-Emission Data. *Journal of Chemometrics*. April 1, 2003, pp 200–215. https://doi.org/10.1002/cem.790.

(6) Drobot, B.; Steudtner, R.; Raff, J.; Geipel, G.; Brendler, V.; Tsushima, S. Combining Luminescence Spectroscopy, Parallel Factor Analysis and Quantum Chemistry to Reveal Metal Speciation - A Case Study of Uranyl(VI) Hydrolysis. *Chem. Sci.* **2015**, *6* (2), 964–972. https://doi.org/10.1039/c4sc02022g.
